# Tuning vision foundation models for rectal cancer segmentation from CT scans

**DOI:** 10.1038/s43856-025-00953-0

**Published:** 2025-07-01

**Authors:** Hantao Zhang, Weidong Guo, Shouhong Wan, Bingbing Zou, Wanqin Wang, Chenyang Qiu, Kaige Liu, Peiquan Jin, Jiancheng Yang

**Affiliations:** 1 School of Computer Science and Technology, University of Science and Technology of China, Hefei, China; 2 Institute of Artificial Intelligence, Hefei Comprehensive National Science Center, Hefei, China; 3 Department of General Surgery, The First Affiliated Hospital of Anhui Medical University, Hefei, China; 4 Department of Radiology, The First Affiliated Hospital of Anhui Medical University, Hefei, China; 5 Anhui Medical University, Hefei, China; 6 Computer Vision Laboratory, Swiss Federal Institute of Technology Lausanne (EPFL), Lausanne, Switzerland

**Keywords:** Rectal cancer, Computed tomography

## Abstract

**Background:**

Rectal cancer segmentation in CT is crucial for timely diagnosis. Despite promising methods, challenges remain due to the rectum’s complex anatomy and the lack of a comprehensive annotated dataset.

**Methods:**

A total of 33,024 slice pairs from 398 rectal cancer patients in a new source center are enrolled into our dataset, named CARE Dataset, with pixel-level annotations for both normal and cancerous rectum tissue. We split it into 317 cases for training and 81 for testing. Additionally, we introduce a segmentation model, U-SAM, which, to the best of our knowledge, is a novel approach designed to handle the complex anatomy of the rectum by incorporating prompt information. Segmentation performance for both normal and cancerous rectum was evaluated using Intersection-over-Union (IoU), Dice Coefficient (Dice), and Normalized Surface Distance (NSD). With the assistance of 46 clinical practitioners, an observer study is conducted to benchmark the U-SAM with human performance and evaluate its clinical applicability. The original new source 398 CT scans and our code are openly available for research.

**Results:**

Our method achieves Dice of 71.23% for normal rectum and 76.38% for rectal tumor, with IoU of 55.32% and 61.78%, and NSD values of 83.63% and 58.59%, respectively, surpassing state-of-the-art methods. The observer study validates that U-SAM can produce diagnostic results comparable to those of highly experienced doctors in just 3 seconds of inference time (with about 5 minutes for prompt acquisition) in clinical settings.

**Conclusions:**

The proposed U-SAM offers an efficient and reliable method for segmenting rectal cancer and normal tissue, significantly reducing time in clinical settings and effectively assisting radiologists. We believe this initial exploration in CT-based rectal cancer segmentation will be instrumental for future diagnosis.

## Plain Language Summary

Establishing the location of tumors from scans taken of people with cancer is helpful for diagnosis and surgical treatment. In the rectum this is challenging due to the rectum structure and the lack of comparative scans from other people. We combined a large number of rectal images from people with cancer to create an image dataset. We also developed a computational method to assist in the identification of normal tissues and areas of rectal cancer. Our work highlights that computational methods can be used to improve the efficiency and accuracy of rectal cancer diagnosis in clinical practice.

## Abstract (web summary)

Zhang et al. construct a large-scale CT image dataset with fine pixel-level annotations for both normal tissue and rectal cancer tumors. Additionally, they propose a clinically applicable method named U-SAM to assist doctors in segmenting normal tissues and rectal cancer areas and validate its effectiveness.

## Introduction

Colorectal cancer ranks as the third most prevalent cancer worldwide and the second leading cause of cancer-related mortality. Notably, rectal cancer accounts for one-third of these cases^1–3^. Timely detection and treatment can effectively halt the further deterioration of the patient’s condition. Clinically, treatment for rectal cancer involves a multidisciplinary approach^1,4,5^, combining surgery with chemoradiation, tailored to the tumor’s size and location, among other factors, to optimize outcomes. Accurate segmentation of both the tumor and the surrounding normal rectal tissue is of significant importance^6^, as it provides valuable imaging anatomical and diagnostic information^7,8^, such as the tumor’s density differences, the extent of tumor invasion into the various layers of the rectal wall, the structure of the serosal surface infiltration, and inflammatory changes in the surrounding tissue. These imaging features offer clinical reference value for radiologists in diagnostic practice^9^. Furthermore, segmenting the relevant areas of the lesion can aid in the development of automated staging networks, which can support AI-based tumor grading systems in extracting corresponding features^10^. This approach helps minimize interference from irrelevant regions, reduces computational load, and enhances model interpretability, potentially improving overall accuracy. On the other hand, it holds significant value for radiotherapy tumor delineation and localization^11,12^. Currently, tumor and organs-at-risk segmentations for radiotherapy are often obtained through labor-intensive manual processes performed by radiation oncologists. AI-assisted accurate segmentations can reduce the workload and support quantitative research.

Among various clinical diagnostic techniques, computed tomography (CT) is preferred for its non-invasive nature, practicality, reliability, and broad applicability in clinical practice. While alternative methods such as endoscopic ultrasound (EUS), positron emission tomography-computed tomography (PET-CT), and magnetic resonance imaging (MRI)^13–15^ are also employed, each has its inherent limitations. EUS, while providing detailed images, is invasive and less favored by patients, particularly due to its limitations in detecting metastatic sites. PET-CT, as a combined functional imaging modality, has lower spatial resolution compared to CT and offers reduced diagnostic accuracy for rectal cancer. It is primarily used for detecting metastases in rectal and other cancers. Moreover, PET-CT is associated with high costs and potential risks due to radiation exposure. MRI technology provides high resolution, and some guidelines (such as those from ACR^16^ and ESGAR^17^) prioritize MRI for the staging of rectal cancer. However, its clinical use is somewhat limited by its high medical costs. In most hospitals in China, CT is still used for the staging diagnosis of rectal cancer^18,19^. Additionally, the prolonged scanning time required for MRI makes it vulnerable to interference from intestinal peristalsis and patient breathing. The longer examination and imaging durations also reduce screening efficiency. Moreover, the fully enclosed environment of MRI scans can cause discomfort for patients with low tolerance, potentially leading to suboptimal imaging results. As a result, MRI is not ideal for routine screening of rectal cancer. Despite these alternatives, this study is based on 64-slice spiral CT scanning technology, which offers advantages such as being non-invasive, practical, convenient, and stable. These characteristics are sufficient to meet the clinical requirements for T-staging^20^, making CT a more commonly used auxiliary method for preoperative staging of rectal cancer and an effective alternative to MRI in settings with limited MRI availability. However, the process of oncologists delineating all colorectal cancer lesions from 3D volumes is both time-consuming and costly^21^.

Deep learning-based medical image segmentation has shown promise in reducing manual delineation efforts, but it necessitates a large-scale finely pixel-level annotated CT image dataset for effective training, particularly in diagnosing specific organ cancerous lesions. Unfortunately, there are currently no datasets available that cover rectal cancer with accurate and detailed annotations due to the extensive time and expertise required for such annotations. Table 1 provides an overview of the mainstream publicly available abdominal datasets, such as BTCV^22^, CHAOS^23^, WORD^24^, and AMOS^25^, which focus on multiple organs in the abdomen. However, these datasets lack diagnostic and lesion information for the rectal region. For specific organ-focused datasets, such as Pancreas-CT^26^ and ACRIN 6664^27^, there is no corresponding cancer lesion information available. Although datasets like FLARE’23^28^, AutoPET^29^, LITS^30^, and MSD^31^ contain lesion information for some organs, they do not include relevant data for the rectum.

**Table 1:** Summary of various publicly available abdominal datasets

Dataset	Modality	Part	Pixel-level	Tumor	Number
BTCV^22^	CT	Abdomen	*✓*	✗	50
CHAOS^23^	CT&MRI	Abdomen	*✓*	✗	80
WORD^24^	CT	Abdomen	*✓*	✗	150
AMOS^25^	CT&MRI	Abdomen	*✓*	✗	600
Pancreas-CT^26^	CT	Pancreas	*✓*	✗	80
ACRIN 6664^27^	CT	Colon	✗	✗	825
FLARE’23^28^	CT	Abdomen	*✓*	*✓*	500
AutoPET^29^	CT&PET	Body	*✓*	*✓*	900
LITS^30^	CT	Liver	*✓*	*✓*	200
MSD^31^	CT	Colon	*✓*	*✓*	190
**CARE(ours)**	**CT**	**Rectum**	*✓*	*✓*	**398**

For datasets that include tumor data, only the count of tumor cases has been compiled. Modality refers to the types of medical data modalities utilized. Part indicates the specific body parts covered by the dataset. Pixel-level denotes whether the dataset includes pixel-level annotations. Tumor specifies whether tumor information is available in the dataset.

The bold text highlights the distinctive characteristics of our CARE dataset compared to existing public datasets. Specifically, it emphasizes that CARE focuses on the rectum, uses CT imaging, provides pixel-level annotations, and importantly includes tumor annotations. This formatting visually underscores the clinical significance of CARE as a large-scale, pixel-annotated CT dataset for rectal cancer.

**Fig. 1:**
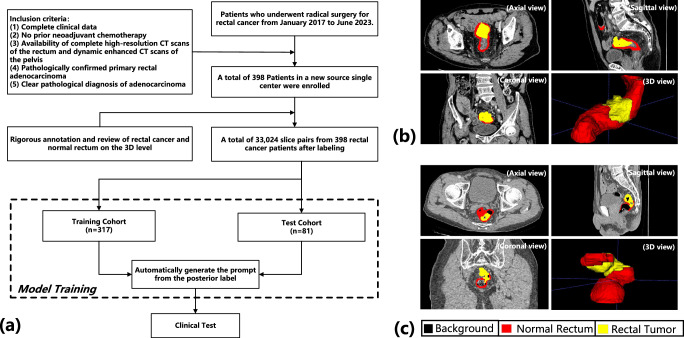
Overview of the CARE dataset construction process and the complex anatomical structure of the rectum. **a** Flowchart of the CARE Dataset setup, including the stages of data collection, model training, and validation through clinical trials. **b** Illustrates the straight section of the rectum. **c** Depicts the curved section of the rectum. Each case of (**b**) and (**c**) highlights segments of a normal rectum and a rectal tumor in CT scans and presents visualizations in axial, sagittal, and coronal views, along with the corresponding 3D rendering results.

Previous studies^22–25,27,28^ emphasized annotating organs rather than cancerous lesions. Although some datasets do include tumor annotations for certain cancer types, regrettably, there is a scarcity of annotated data specifically covering rectal cancer regions. In this study, we aim to address the gap in the field of rectal cancer segmentation by collecting a large-scale real clinical rectal-cancer CT image dataset with careful pixel-level annotation. Examples of CT scans and annotations from the CARE (Clinical Annotation for REctal cancer segmentation) are illustrated in Fig. 1b, c. CARE obtains fine-grained annotations for both normal rectal regions and diseased tumor regions. More visualizations of different morphology samples for the CARE dataset can be found in Supplementary Figs. 1–3. Collecting real clinical data poses significant challenges due to the difficulty in harmonizing source data with medical expertise and formatting. Besides, it demands ethical protection due to privacy concerns. Moreover, annotating a large-scale medical image segmentation dataset, especially for rectal cancer, is a costly and labor-intensive endeavor, necessitating much domain knowledge and clinical experience. In each case, a panel of experienced doctors, with more than 20 years of expertise, engage in thorough discussions to precisely identify the location and margins of the rectal cancer. Once a consensus is reached, detailed pixel-level annotations are meticulously crafted by one of them. Overall, CARE is the first large-scale real clinical rectal cancer segmentation dataset from CT images. All images in CARE were anonymized and approved by the ethics committee to protect privacy, with all clinical treatment details removed. It will soon be publicly available.

Currently, mainstream medical segmentation models focus on large organs. However, cancer tumors often have complex morphological characteristics that differ significantly from larger organ structures. Previous methods perform poorly in rectal cancer segmentation due to insufficient relevant medical prompt information. To solve these problems, We propose a interactive segmentation system named U-SAM (U-shaped SAM) to assist doctors in segmenting normal intestinal walls and rectal cancer areas. Compared to current medical image segmentation methods, U-SAM allows doctors to integrate prior medical knowledge interactively. The observer study with 46 clinical practitioners validated that U-SAM can achieve diagnostic results comparable to highly experienced doctors in just 3 seconds of inference time. This significantly improves the efficiency and accuracy of rectal cancer diagnosis in clinical practice.

Briefly, we make the following contributions in this paper:

First, we construct a large-scale CT-image dataset for rectal-cancer segmentation. To the best of our knowledge, our dataset CARE is the first large-scale CT image dataset with fine pixel-level annotations for the lesion information of rectal cancer. Several state-of-the-art segmentation methods are evaluated on the CARE dataset. The images within the CARE dataset are anonymized and approved by the ethics committee. The dataset is scheduled to be publicly available soon.

Second, driven by the SAM’s innovative promptable segmentation paradigm, we develop a prompting model named U-SAM for rectal cancer segmentation. The model incorporates the convolutional U-shaped adapter designed to capture localized lesion information. Furthermore, we introduce an innovative variant of skip-connection between U-shaped adapter to enhance the decoder’s segmentation performance. Extensive experiments demonstrate that the proposed U-SAM outperforms state-of-the-art methods on the CARE and WORD datasets.

Finally, to validate the clinical efficacy of our U-SAM model, we conduct a study with 46 clinical practitioners. The observer study confirms that U-SAM achieves diagnostic results comparable to highly experienced doctors in just 3 s of inference time.

## Materials

### Overview

The CARE rectal cancer dataset comprises CT scans from 398 patients, all diagnosed with rectal cancer. The original CT data and annotation files together exceed 74 GB in size. To ensure the imaging quality, all CT data has undergone enhancement. For a more accurate assessment of the tumor’s condition, the patients providing the samples received corresponding contrast agents via blood vessel injection. This allows for easy identification of the rectal cancer area through changes in the contrast agent within the lesion and the mass enhancement. Each case in our dataset is backed by corresponding postoperative histopathological slides and analyses. It is important to note that all CT images are anonymized. All clinical treatment details have been removed. The CARE dataset required around two years of meticulous collection, annotation, and rigorous review. This extensive effort has resulted in a valuable resource that bridges the gap in the field of rectal cancer segmentation.

### Ethics

This study was approved by the Ethics Committee of the First Affiliated Hospital of Anhui Medical University (No. Quick-PJ 2023-13-34). As this is a retrospective study and data analysis was performed anonymously, with all treatment details removed, it was deemed unnecessary to obtain informed consent from patients. All procedures involving human participants in this research were in accordance with Chinese national ethical standards.

### Inclusion criteria

This study retrospectively analyzed 398 cases of rectal cancer patients who underwent radical surgery at the First Affiliated Hospital of Anhui Medical University from January 2017 to June 2023. A 64-slice spiral CT scanner (Revolution CT; GE Medical Systems, Illinois, USA) was used for CT scanning from the abdomen to the pelvis. An intravenous injection of 80 ml of iodine contrast (Omnipaque; GE Healthcare, Shanghai, China) was administered, and enhanced scanning was performed within 30 s thereafter. Images from the portal venous phase were selected for the construction of the dataset. Each CT scan utilized the following acquisition parameters: a slice thickness of 0.625 mm or 1.25 mm, reconstruction interval of 0.625 mm or 1.25 mm, tube voltage of 120 kVp, tube current of 8–420 mA, high-resolution matrix size of 512 × 512, field of view of 500 mm, rotation time of 0.75 s, pitch of 0.984, and pixel size of 1.46 mm. During the portal venous phase, the tumor lesions were significantly enhanced, facilitating the distinction between the tumor area and the surrounding normal tissue. Many previous studies have also utilized enhanced CT images from the portal venous phase to segment tumor lesions^32–34^.

As detailed in Fig. 1a, the inclusion criteria are as follows: complete clinical data, no prior neoadjuvant chemotherapy, availability of complete high-resolution CT scans of the rectum and dynamic enhanced CT scans of the pelvis, pathologically confirmed primary rectal adenocarcinoma, and clear pathological diagnosis of adenocarcinoma.

### Professional data annotation

The CARE dataset undergoes a rigorous annotation and review process to ensure its reliability. Initially, a gastrointestinal surgery clinician and a radiologist(both possessing over 20 years of experience) collaboratively analyze the patient’s condition and the location of rectal cancer. Subsequently, more than ten gastrointestinal surgeons with extensive clinical expertise utilize ITK-SNAP^35^ to delineate the diseased rectal area and the normal rectum slice-by-slice according to comprehensive information on different axial views. Finally, two oncology experts with over 20 years of experience thoroughly examine and revise these annotations. In cases of disagreement, they engage in discussions to reach a consensus on annotations, further enhancing the overall annotation quality. Annotation details is available in Supplementary Note 6.

### Data construction of CARE dataset

We conducted a random split of the CARE dataset into two subsets: 318 cases for training and 81 cases for testing. We also took the necessary steps to enhance training efficiency by eliminating irrelevant regions. Slices not containing the rectum were removed, and the corresponding images and labels were then packed into image-label pairs. In the end, we obtained 26,563 slice pairs for training and 6461 pairs for testing. A detailed breakdown of the dataset into training and testing cohorts is provided in Supplementary Table 1, including comprehensive clinical baseline pathology data of 398 patients. This includes the distribution of gender, age, tumor characteristics, levels of biomarkers, and other relevant physiological measurements^36^. The data are segmented into total, training, and testing cohorts, facilitating a comprehensive comparison and analysis.

## Methods

### Overview

**Fig. 2:**
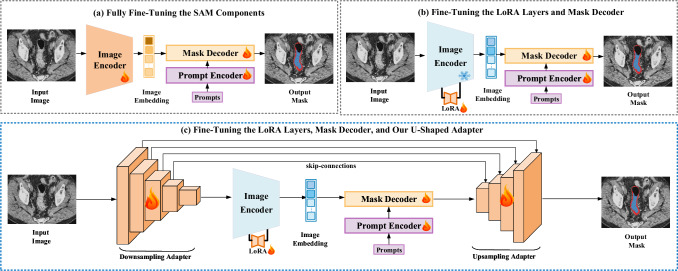
Pipeline comparison between the traditional fine-tuning method of Segment anything model (SAM) and our U-shaped adapter. **a** Fully fine-tuning the SAM component, including the image encoder, prompt encoder, and mask decoder, as discussed in refs. ^37–39^. **b** Fully fine-tuning the prompt encoder and mask decoder, freezing the image encoder, and inserting additional trainable LoRA layers^57^ as per ref. ^40^. **c** Fully fine-tuning the prompt encoder and mask decoder, freezing the image encoder, and inserting additional trainable LoRA layers and the U-shaped adapter.

Figure 2c illustrates the overview of our U-SAM framework. To the best of our knowledge, current SAM-based segmentation methods^37–40^ mainly focus on transferring learning knowledge from natural and medical images without modifying too much architecture of the based SAM model shown as Fig. 2a. No efforts have been directed toward modifying the intrinsic architecture of SAM to align it more effectively with the requirements of the medical domain. In this study, we propose a architecture named U-SAM designed to enhance the segmentation capability within the medical domain. We integrate the promptable paradigm into U-SAM to enhance lesion localization and capture intricate details more effectively. Specifically, U-SAM contains three key components: promptable information (e.g., points) to aid in target area localization, U-shaped for capturing low-level lesion details, and skip-connections to preserve and recover spatial information during the encoding-decoding process.

In the context of rectal cancer segmentation, achieving optimal results can be pretty challenging for conventional segmentation models(e.g., MissFormer^41^, TransUnet^42^, SwinUnet^43^, UCTransNet^44^). This difficulty arises from the irregular shape of the rectum and the delicate nature of some intestinal walls, which possess thin wall thickness, as illustrated in Fig. 1b, c. These unique anatomical characteristics often pose hurdles to accurately segmenting the rectal region. Inspired by the success of Segment anything model’s (SAM)^45^ innovative promptable segmentation paradigm(e.g., bounding boxes, points, texts), we aim to introduce this paradigm to enhance the localization of the target rectum and address the intricate challenges posed by the complex nature of the rectal region. More discussions of interactive medical segmentation can be found in Supplementary Note 2. However, the SAM model struggles to attain optimal segmentation in scenarios involving low contrast and intricate tissue structures due to constraints in detail extraction^37^. This challenge is particularly critical in the context of rectal-cancer segmentation.

#### SAM for medical imaging

To tackle the above challenges, we propose a model named U-SAM (U-shaped SAM), as depicted in Fig. 2c. Figure 2 shows the currently mainstream fine-tuning methods for the SAM in the field of medical domain. Instead of solely relying on fine-tuning the SAM model^37–39^ as Fig. 2a, we leverage SAM’s promptable paradigm and integrate prior medical knowledge to develop a segmentation model. We add the U-shaped adapter to the beginning and end of the original SAM model, including a downsampling adapter and an upsampling adapter. In U-SAM’s image encoder, the Transformer encodes tokenized image patches from a convolutional neural network (CNN) feature map, serving as the input sequence to extract additional lesion details. To reduce the number of parameters during the training process, the image encoder can be frozen, with additional LoRA layers inserted for training shown as Fig. 2b. As for U-SAM’s decoder, it performs upsampling on the encoded features, which are then fused with high-resolution CNN feature maps to produce more precise segmentation masks.

### U-SAM framework

Following the framework of the original SAM, our U-SAM generally utilizes a two-step upsampling scheme to reconstruct the resolution. Instead of the aggressive long-stride upsampling strategy adopted in original SAM, we introduce a pair of U-shaped adapters in the U-SAM.

The framework of U-SAM is shown in Fig. 2c. The pipeline of U-SAM can be generally divided into two major processes, including the downsampling encoder and upsampling decoder. In the following discussion, the image encoder is viewed as part of downsampling, and the mask decoder is defined as the beginning of upsampling.

#### Downsampling adapter

In the downsampling encoder of our U-SAM, we extract feature representation using four consecutive downsampling blocks, each decreasing the resolution of the feature map by half. The process can be formulated as:

1 $${f}_{i+1}=Conv(MaxPool(\,{f}_{i}))$$

where *f*_*i*_ and *f*_*i*+1_ indicate the input and output feature of the *i*^*th*^ downsampling block, correspondingly. *MaxPool* and *Conv* represents 2-D max pooling module and 2-D convolution module with a kernel size of 3, respectively.

As depicted in Fig. 2c, we obtain feature maps from all five layers. These are denoted as ${f}_{0}\in {{\mathbb{R}}}^{\frac{C}{8}\times H\times W}$, ${f}_{1}\in {{\mathbb{R}}}^{\frac{C}{4}\times \frac{H}{2}\times \frac{W}{2}}$, ${f}_{2}\in {{\mathbb{R}}}^{\frac{C}{2}\times \frac{H}{4}\times \frac{W}{4}}$, ${f}_{3}\in {{\mathbb{R}}}^{C\times \frac{H}{8}\times \frac{W}{8}}$, and ${f}_{4}\in {{\mathbb{R}}}^{3C\times \frac{H}{16}\times \frac{W}{16}}$. Here, *C* = 256 represents the channel dimension of the latent space in the Spatial Attention Module (SAM). The feature representation *f*_4_ is subsequently input into SAM’s image decoder, while *f*_0_, *f*_1_, *f*_2_, and *f*_3_ are integrated into the upsampling decoder process via skip connections.

#### Upsampling adapter

In the SAM architecture, given the high-level feature representation *f*_4_ and the prompt embedding *pe*, the mask decoder generates raw mask data, including the mask source $Src\in {{\mathbb{R}}}^{C\times \frac{H}{16}\times \frac{W}{16}}$ and mask tokens $Mt\in {{\mathbb{R}}}^{N\times C}$. The transformation of *Mt* to $M{t}^{{\prime} }$ follows the procedure established in the original SAM. However, deviating from the original methodology, U-SAM implements a sequence of three 2 × upsampling blocks—namely *UP*^4^, *UP*^3^, and *UP*^2^—to refine *Src* into $Sr{c}^{{\prime} }\in {{\mathbb{R}}}^{\frac{C}{8}\times \frac{H}{2}\times \frac{W}{2}}$. The corresponding upsampling operations are expressed mathematically as:

2 $${r}_{i}=U{P}^{i+1}({r}_{i+1},{f}_{i+1}),\quad i=1,2,3$$

In this equation, *r*_*i*+1_ and *r*_*i*_ represent the incoming and outgoing source features, respectively, with *f*_*i*+1_ indicating the image feature map transmitted via skip connection. Contrasting with the original SAM, which employs 4 × bilinear interpolation for upsampling low-resolution logits *l*, the enhanced U-SAM incorporates an additional 2 × upsampling block, labeled *UP*^1^. This block is utilized to fully upscale the logits *L*, guided by the skip-connected feature *f*_0_.

### Implementation details

In all experiments, we utilized PyTorch to implement our model, leveraging 8 NVIDIA 3090 GPU cards, each equipped with 24 GB of memory. To prevent overfitting, we applied two types of fundamental online data augmentations: random flipping and random rotating. It’s worth noting that all of our experiments are based on the SAM-ViT-B. Additionally, we utilized the pre-trained weights of SAM^45^ on natural images to expedite convergence and enhance training stability. Following the previous work^42,43^, we set the input resolution to 224  × 224. The batch size is used as 24. Our method is trained in an end-to-end manner, employing the Adam optimizer^46^. To expedite faster convergence, the initial learning rates for the encoder and decoder parts are set to 0.001 and 0.0001, respectively. We also employ the combined cross entropy loss and dice loss as our loss function to train our network. We followed the guidelines from Metrics Reloaded^47^, while also considering related works^37,48^, and employed the Dice similarity coefficient (Dice), Intersection over Union (IoU), and normalized surface distance (NSD)^49^ to quantitatively assess segmentation performance on the CARE dataset. Additionally, we reported the Dice scores for all organs in the WORD dataset. More implementation details for the experiments are provided in Supplementary Note 8.

### Reporting summary

Further information on research design is available in the Nature Portfolio Reporting Summary linked to this article.

## Results

### Comparison with *State of the Art*

To demonstrate the effectiveness of our proposed U-SAM, we compare our model with current state-of-the-art methods. We cover two types of methods for the comprehensive evaluation, including conventional segmentation methods (e.g., MissFormer^41^, TransUnet^42^, SwinUnet^43^, UCTransNet^44^) and promptable paradigm model containing promptable information (e.g., SAM^45^, SAM+LoRA^40^). All experimental performance results are reported based on the test set. The results are presented in Table 2, with the best outcomes highlighted in bold.

**Table 2:** Comparisons of performance with existing methods on the CARE dataset

Method	Normal			Tumor			Mean		
	Dice (%)	IoU (%)	NSD (%)	Dice (%)	IoU (%)	NSD (%)	Dice (%)	IoU (%)	NSD (%)
AttenUnet^60^	62.88	45.86	73.23	71.16	55.23	51.47	67.02	50.54	62.35
ResUnet++^61^	59.61	42.46	69.22	68.55	52.15	47.62	64.08	47.30	58.42
MultiResUnet^62^	62.25	45.19	74.31	72.54	56.92	51.75	67.40	51.05	63.03
MissFormer^41^	57.79	40.63	68.25	69.02	52.69	45.01	63.40	46.66	56.63
SwinUnet-B^43^	63.32	46.32	76.15	72.63	57.02	54.31	67.97	51.67	65.23
SwinUnet-L^43^	61.66	44.57	73.62	72.58	56.97	52.32	67.12	50.77	62.97
TransUnet-B^42^	60.74	43.62	73.48	70.65	54.62	51.35	65.70	49.12	62.42
TransUnet-L^42^	63.75	46.79	76.34	72.60	56.98	54.14	68.17	51.86	65.24
UCTransNet^44^	63.00	45.98	74.56	72.90	57.35	54.19	67.95	51.67	64.38
nnUNet^63^	57.23	40.92	57.73	71.92	57.38	45.11	64.58	49.15	51.42
SAM^45^	60.95	43.83	75.31	71.00	55.04	52.79	65.98	49.44	64.05
SAM+LoRA^40^	57.57	40.42	64.97	70.70	54.68	47.16	64.14	47.55	56.07
**U-SAM(Ours)**	65.72	48.94	77.98	72.84	57.28	52.60	69.28	53.11	65.29
**U-SAM+LoRA (Ours)**	64.24	47.32	76.41	72.50	56.86	52.31	68.37	52.09	64.36
**U-SAM/P(Ours)**	70.27	54.17	83.42	**76.49**	**61.94**	**59.81**	73.38	58.05	**71.62**
**U-SAM/B(Ours)**	**71.23**	**55.32**	**83.62**	76.38	61.78	58.59	**73.80**	**58.55**	71.11

‘B’ denotes that the model utilizes the ‘ViT-B’, while ‘L’ indicates the ‘ViT-L’. For the SAM-based model, we all utilized SAM-B^45^. ‘U-SAM/P’ refers to the model incorporating 3 points prompt per class. ‘U-SAM/B’ refers to the U-SAM model utilizing the box-based prompts. The entries in bold represent the best performances achieved on the CARE dataset.

Similar experimental results are obtained to the^38^, relying solely on fine-tuning the basic SAM model, falls behind the specialized segmentation models. Moreover, while keeping the pre-trained weights locked, the approach of exclusively fine-tuning the adapter, as demonstrated in ‘SAM+LoRA’^40^, also faces challenges in attaining ideal results. Rather than solely on transfer learning, we propose a U-shaped adapter architecture to better adapt to the medical domain. Even without adding any promotable information, our proposed U-SAM outperforms all other competing methods, which validates the superiority of U-shaped adapter architecture. Furthermore, thanks to the promotable paradigm, U-SAM obtains excellent results when adding 3 points promptable information per class. Specifically, ‘U-SAM/P’ achieves a 5.21% mean Dice, 6.19% mean IoU and 6.38% mean NSD gain over the state-of-the-art methods. When employing box-based prompts, ‘U-SAM/B’ also shows further enhancement, yielding a 5.63% mean Dice, 6.69% mean IoU, 5.87% mean NSD gain than the current state-of-the-art methods. We also implement a low-computation version of U-SAM, denoted as ‘U-SAM+LoRA’ in Table 2. This model still achieves satisfactory results.

We further explore the generalization of U-SAM on the WORD^24^ dataset, employing two types of evaluation methods similar to those used for the CARE dataset. The experimental results once again demonstrate U-SAM’s excellent performance, highlighting our model’s remarkable ability to generalize and its versatility compared to the traditional specialized segmentation models. Comparisons of performance with existing methods on the WORD dataset in Supplementary Note 4.

### Observer study

#### Setting

To further validate the clinical efficacy of our proposed U-SAM model, we assembled a cohort of 46 clinical practitioners as experimental participants, none of whom had been involved in the construction of the CARE dataset. Based on the clinical experience and qualifications of these doctors, they were divided into three groups: Group 1 with 15 participants, Group 2 with 14 participants, and Group 3 with 17 participants. Group 1 consists of novice doctors who can adequately identify colorectal tumors in clinical settings. Group 2 includes doctors with some years of surgical clinical experience. Group 3 comprises highly experienced clinicians who can diagnose colorectal tumors both quickly and accurately. Besides, we extracted twenty rectal cancer patient CT scans from the CARE test set to serve as clinical trial test cases. Each of the 46 doctors cyclically annotated these 20 CT scans to ensure that each case was annotated at least ten times.

To facilitate the clinicians’ annotation process during the experiment, we provided the doctors with the starting and ending frames of the regions requiring annotation for each case. Within these specified segments, the doctors annotated the normal rectal walls and tumors.

#### Human performance

Ultimately, we collected annotation results for 297 cases from three groups of doctors. The data showed that, on average, each case was annotated 14.85 times, with Group one annotating each sample 4.85 times, Group two 4.9 times, and Group three 5.1 times. We recorded the time each participating doctor spent on the annotations and assessed their accuracy.

##### Time cost

The time required by different doctors to diagnose and annotate a patient case is shown in Supplementary Fig. 7. The figure illustrates that the annotation time for patient cases varies significantly among doctors, influenced by individual clinical experience and other factors. Generally, novice doctors (Group 1) tend to spend more time annotating cases compared to those with some clinical experience (Group 2 and Group 3). The box plots also indicate that novice doctors (Group 1) exhibit greater annotation time variability than Groups 2 and 3.

##### Accuracy

The diagnostic and annotation accuracy of doctors under different conditions is shown in Supplementary Fig. 8. We computed the dice scores for various annotated cases and provided the dice scores of our different model variants for the test cases. Diagnostic and annotation accuracy among different doctors often varies significantly for the same case, a phenomenon particularly noticeable among less experienced doctors, as shown in Supplementary Fig. 8, Group 1. More experienced doctors tend to achieve greater consistency and overall accuracy in diagnosing and annotating the same case, as illustrated in Supplementary Fig. 8, Group 3.

We further analyzed and compared the efficiency and accuracy between clinicians and our proposed U-SAM model. Table 3 presents the average time required to annotate a case by different categories of doctors and various versions of the U-SAM model, alongside their corresponding average accuracy. The average time taken to diagnose and annotate a case varies according to clinical experience: Group 1 requires 166 min, Group 2 requires 141 min, and Group 3 requires 111 min. In contrast, our proposed U-SAM model requires only around three seconds for inference. The numbers in parentheses in Table 3 reflect the additional time clinicians require to annotate prompts. In general, point-based prompt annotation takes less time than box-based annotation, ~3 min versus 5 min, respectively. However, the time required for manual annotation can vary significantly depending on user habits and how the model is applied, making direct comparisons difficult. In terms of segmentation accuracy, our proposed U-SAM can achieve a diagnostic and segmentation accuracy comparable to that of mid-level doctors (Groups 1 and 2) without relying on any prompt information. When point and box prompts are incorporated, the segmentation accuracy improves further. U-SAM/B, using box prompts, surpasses the accuracy of highly experienced doctors (Group 3), achieving the highest average Dice, IoU and NSD scores.

**Table 3:** Comparisons of efficiency and accuracy between clinicians and our proposed U-SAM

Method	Time	Normal			Tumor			Mean		
		Dice (%)	IoU (%)	NSD (%)	Dice (%)	IoU (%)	NSD (%)	Dice (%)	IoU (%)	NSD (%)
Group 1	166min	54.67	42.83	53.88	68.10	48.50	40.65	61.38	45.67	47.27
Group 2	141min	51.51	39.07	52.01	65.76	45.94	35.48	58.63	42.50	43.74
Group 3	111min	61.48	49.27	58.62	**74.63**	54.32	46.30	68.06	51.80	52.46
U-SAM	3s	61.86	46.29	73.21	64.62	49.58	45.14	63.24	47.94	59.18
U-SAM/P	3s (+3min)	66.24	51.19	78.50	70.35	55.34	**51.14**	68.29	53.26	64.82
U-SAM/B	3s (+5min)	**67.73**	**52.23**	**79.24**	69.72	**54.77**	50.90	**68.72**	**53.50**	**65.07**

The table displays the average time taken to annotate a case by different categories of doctors (Group 1, Group 2, and Group 3) and various versions of the U-SAM model, along with their corresponding average annotation accuracy. For the SAM-based model, we all utilized SAM-B^45^. U-SAM/P refers to the model incorporating 3 points prompt per class. U-SAM/B refers to the U-SAM model utilizing the box-based prompts. The entries in bold represent the best performances.

## Discussion

The automatic segmentation of rectal cancer tumors and normal rectal tissue in medical imaging is of significant value for the automated staging diagnosis of rectal cancer. Recent research efforts^50–53^ have aimed to segment the normal intestinal wall and potential tumors around the colorectum in conventional CT scans. However, these studies primarily focused on traditional segmentation paradigms and have not incorporated interactive segmentation methods^37^ to handle the complexities of hollow viscera. Topologically, the rectum and colon are distinct anatomical regions; the colorectum^52^ has a single-path and continuous structure extending between the caecum and the rectum. Additionally, compared to the surrounding tissues and organs involved in colon cancer^54,55^, rectal cancer, especially mid and low rectal cancer, involves a more complex pelvic floor structure and has the potential to invade the pelvic floor muscles.

In this context, deep-learning-assisted segmentation of rectal cancer using U-SAM is significant in several ways. First, in hierarchical medical care, high-level hospitals can leverage their abundant resources and clinical expertise to assist lower-level hospitals in delivering more efficient and effective diagnoses and treatments. Second, manual segmentation of both the tumor and the surrounding normal rectal tissue is labor-intensive, time-consuming, and inefficient^56^. AI tools such as U-SAM can reliably analyze medical images, better aligning with the consensus views of experienced medical professionals to provide more stable diagnostic outcomes. By reducing segmentation time, these tools offer a consistent and solid basis for diagnosis, enhancing the overall efficiency and reliability of medical assessments. For junior and mid-level physicians (e.g., Group 1 and Group 2), such tools can help reduce the occurrence of minor errors to some extent, while improving the quality and speed of medical image analysis. For senior physicians (e.g., Group 3), U-SAM effectively reduces repetitive tasks by quickly identifying relevant tumor regions through highlighted points or bounding box information, thereby saving segmentation time.

**Fig. 3:**
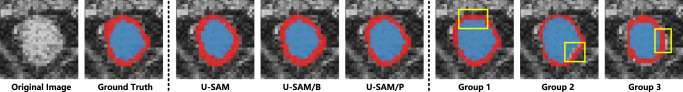
The qualitative comparison between clinicians and our proposed U-SAM. Red indicates normal rectal tissue, while the blue represents rectal cancer tumors. The yellow boxes highlight the regions where the model makes mistakes compared to the ground truth.

**Fig. 4:**
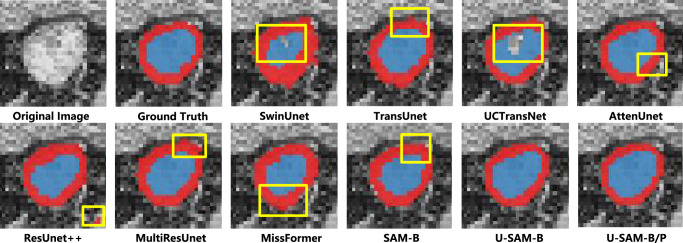
The qualitative comparison on the CARE dataset. Red indicates normal rectal tissue, while the blue represents rectal cancer tumors. The yellow boxes highlight the regions where the model makes mistakes compared to the ground truth.

To assess the model’s practical performance, Fig. 3 compares the diagnostic and segmentation annotations of normal rectal walls and tumors made by our proposed U-SAM model with those made by clinicians. Notably, the boxes highlight the regions where the model makes mistakes compared to the ground truth. The figure shows that artificial intelligence can more accurately delineate the boundaries between tumors and healthy rectal walls than clinical assessments. Additionally, AI-assisted rectal cancer segmentation is more consistent than clinical evaluations. Even for the same case, various groups of doctors (Group 1, Group 2, and Group 3) often reach different conclusions, as shown in Fig. 3. We further visualize the segmentation results of the comparable models in Fig. 4. It shows that our U-SAM generates better segmentation results, which are more similar to the ground truth than the baseline model results. It shows that our proposed method excels in accentuating salient areas while eliminating perplexing false positive lesions and producing coherent boundaries. These insights imply that U-SAM can achieve more refined segmentation while preserving intricate shape information, particularly along the borders of the normal rectum and tumor regions, as illustrated in Fig. 4. We also visualize the irregular anatomical structure segmentation results on the CARE dataset. More details can be found in Supplementary Note 1. These visual comparisons validate U-SAM’s potential as a reliable clinical aid and motivate further investigation into its internal components and architectural advantages.

To better understand the model’s internal components, we conduct ablation experiments to demonstrate U-SAM’s superiority in U-shaped adapter architecture and promptable (e.g., points) segmentation paradigm on the CARE dataset. To enhance the reliability of the experiment, we conducted each trial five times and reported the corresponding mean and standard deviation (mean  ± s.d.). As shown in Table 4, the best mean values are highlighted in bold. Due to SAM’s inherent structural limitations, it does not yield optimal results in the medical domain. However, by incorporating a convolutional module and connecting the encoder and decoder with skip-connections to form a U-shaped model, we achieved a relative improvement of 2.1% in mean Dice, 2.35% in mean IoU, and 1.78% in mean NSD.

**Table 4:** Ablation experiments on the CARE dataset

Method	Normal			Tumor			Mean		
	Dice (%)	IoU (%)	NSD (%)	Dice (%)	IoU (%)	NSD (%)	Dice (%)	IoU (%)	NSD (%)
S	61.37 ± 0.27	44.27 ± 0.28	74.76 ± 0.29	71.58 ± 0.38	55.74 ± 0.47	51.74 ± 0.77	66.48 ± 0.26	50.01 ± 0.30	63.25 ± 0.49
S+U	64.23 ± 0.80	47.31 ± 0.87	76.57 ± 0.81	72.93 ± 0.59	57.40 ± 0.73	53.49 ± 1.16	68.58 ± 0.54	52.36 ± 0.62	65.03 ± 0.73
S+U+1 point	66.62 ± 0.92	49.95 ± 1.03	79.46 ± 0.32	74.83 ± 0.46	59.78 ± 0.59	56.99 ± 1.03	70.72 ± 0.39	54.87 ± 0.44	68.22 ± 0.59
S+U+3 points	69.61 ± 0.35	53.39 ± 0.41	**82.85** **±** **0.33**	**75.75** **±** **0.42**	**60.97** **±** **0.54**	**57.71** **±** **1.10**	**72.68** **±** **0.37**	**57.18** **±** **0.45**	**70.28** **±** **0.68**
S+U+5 points	**69.65** **±** **0.62**	**53.44** **±** **0.74**	82.30 ± 0.21	75.59 ± 0.50	60.76 ± 0.64	56.23 ± 1.59	72.62 ± 0.31	57.10 ± 0.37	69.27 ± 0.90

‘S’ denotes the Baseline(SAM). ‘U’ represents the use of a U-shaped adapter, incorporating convolutional modules and skip connections to create a U-shaped network. All of the results, including the mean and standard deviation (mean ± s.d.) across five trials. The entries in bold represent the best performances.

Beyond structural improvements, we also investigate the role of prompt-based interaction in enhancing segmentation performance. We further investigate the potential of U-SAM’s point-prompted segmentation paradigm. With a single anchor point per class, the ‘S+U+1 point’ configuration achieves improvements of 2.14% in mean Dice, 2.51% in mean IoU, and 3.19% in mean NSD. Increasing the points to three per class, the ‘S+U+3 points’ setup yields the best performance, with improvements of 4.10% in mean Dice, 4.82% in mean IoU, and 5.25% in mean NSD. However, despite the random sampling of promptable points, increasing the number of points does not consistently enhance performance. As such, ‘S+U+5 points’ fails to provide further gains, leading us to adopt the use of three points in all subsequent optimal configurations. We also performed one-sided t-tests on the three mean metrics to compare the configurations ‘S’ vs. ‘S+U’ and ‘S+U’ vs. ‘S+U+3 points’. The *p*-values for all comparisons were well below the significance threshold of 0.05, confirming that the improvements from both the U-shaped adapter and the prompt information are statistically significant.

**Fig. 5:**
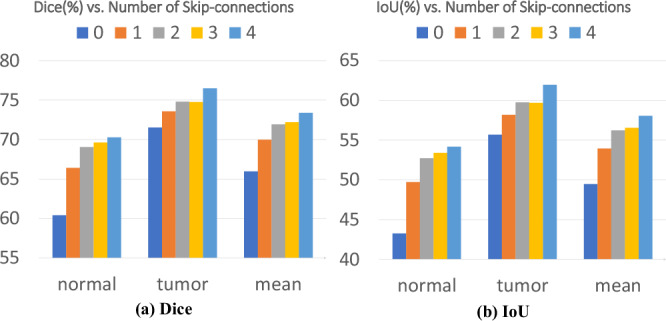
Effect of skip-connections on U-SAM performance. Ablation study results showing the impact of skip-connections on U-SAM performance: **a** Dice across models with different numbers of skip-connections; **b** IoU across models with different numbers of skip-connections.

Following the analysis of structural design and prompt-based interactions, we next investigate the impact of skip-connections within the U-SAM architecture. As previously explained, incorporating U-Net-like skip-connections within the U-SAM architecture plays a crucial role in retaining and restoring spatial information throughout the encoding-decoding process. Consequently, we conduct an ablation study concerning the number of skip-connections to ascertain the optimal configuration for these connections. All experiments in this section utilize 3 points per class. Specifically, we vary the number of skip-connections from 0 to 4 and conduct all experiments on the CARE dataset. It should be noticed that ‘0-skip’ uses no skip-connections, ‘1-skip’ integrates a sole skip-connection at a scale of $\frac{1}{8}$, while ‘2-skip’ implements two skip-connections, strategically positioned at both $\frac{1}{8}$ and $\frac{1}{4}$ scales, and so forth. The experiment results for Dice and IoU are visually represented in Fig. 5. The results show that adding more skip-connections generally leads to a better segmentation performance. The best mean Dice and mean IoU are achieved by inserting skip-connections to all four intermediate upsampling steps of $\frac{1}{8}$, $\frac{1}{4}$, $\frac{1}{2}$ and 1 resolution scales. Thus, this best configuration is adopted in our U-SAM. It is also worth mentioning that significant enhancements have been attained for both normal rectum and tumor. This indicates the effectiveness of integrating U-Net-like skip-connections within the SAM framework, which benefit the extraction of intricate details, such as the precise delineation of the rectum’s borders.

In addition to segmentation performance, we also examine the computational efficiency of U-SAM to assess its practical deployment potential. We present a detailed analysis of the performance of various model variants on the CARE and WORD datasets. In all our comparative experiments, we evaluate the performance of SAM-based models without utilizing any prompt information (e.g., points, box). As depicted in Table 5, our proposed U-SAM consistently outperforms mainstream medical segmentation methods (e.g., UCTransNet^44^, TransUnet^42^), while consuming fewer computational resources on the CARE dataset. Compared to the SAM(e.g., SAM-B), with 90.21 million parameters and 17.08 GFLOPs, our proposed U-SAM is just slightly higher, with 103.36 million and 25.07 GFLOPs. This marginal increase is primarily attributed to the incorporation of U-shaped adapter structures in both the encoder and decoder. Simultaneously, U-SAM demonstrates a remarkable improvement of 3.3% in mean Dice and 3.67% in mean IoU. We also offer a comparison with ‘SAM+LoRA’^40^ by implementing the U-SAM using Low-Rank Adaptation^57^ to ensure a fair assessment. ‘SAM+LoRA’^40^ freezes the image encoder and only fine-tunes by inserting additional trainable LoRA layers. As a result, ‘SAM+LoRA’^40^ has a relatively low number of tunable parameters. However, only fine-tuning LoRA layers is insufficient, as it falls behind mainstream specialized medical image segmentation methods in segmentation performance. In contrast, our proposed U-SAM+LoRA introduces a U-shaped adapter to address this issue. By adding only a small number of parameters, it achieves relatively better segmentation performance. Our evaluation also extends to the WORD dataset, and consistent experimental results are observed. For more in-depth details, please refer to Supplementary Note 5.

**Table 5:** Comparison of parameters, tunable parameters, GFLOPs, mean Dice, mean IoU, and NSD with state-of-the-art medical segmentation methods on the CARE Dataset

Method	Param (M)	TParam (M)	GFLOPs	Dice (%)	IoU (%)	NSD (%)
UCTransNet^44^	66.24	66.24	32.93	67.95	51.67	64.38
TransUnet-B^42^	93.23	93.23	24.68	65.70	49.12	62.42
SwinUnet-B^43^	149.11	149.11	30.25	67.97	51.67	65.23
TransUnet-L^42^	315.08	315.08	68.17	68.17	51.86	65.24
SwinUnet-L^43^	335.26	335.26	67.96	67.12	50.77	62.97
SAM-B^45^	90.21	90.21	17.08	65.98	49.44	64.05
SAM-B+LoRA^40^	90.36	3.93	17.11	64.14	47.55	56.07
U-SAM-B	103.36	103.36	25.07	69.28	53.11	65.29
U-SAM-B+LoRA	103.51	17.68	25.10	68.37	52.09	64.36
SAM-L^45^	307.87	307.87	59.74	67.07	50.70	63.23
SAM-L+LoRA^40^	308.26	4.32	59.82	65.09	48.51	60.70
U-SAM-L	325.40	325.40	68.60	69.06	52.93	64.98
U-SAM-L+LoRA	325.79	22.64	68.65	68.68	52.07	64.58
SAM-H^40^	635.35	635.35	123.90	NaN	NaN	NaN
SAM-H+LoRA^40^	636.01	4.44	124.03	65.38	48.80	61.04
U-SAM-H	658.73	658.73	133.88	NaN	NaN	NaN
U-SAM-H+LoRA	659.69	28.81	134.02	68.95	52.74	65.72

‘B’ denotes that the model utilizes ‘ViT-B’, while ‘H’ indicates ‘ViT-H’. ‘TParam’ represents the tunable parameters. ‘LoRA’ refers to the model implemented using Low-Rank Adaptation^57^. ‘NaN’ indicates that the value exceeds the evaluative capacity of our computational resources.

While our results demonstrate strong performance across multiple aspects, several limitations remain. This study focuses on CT as the imaging modality due to its widespread use in clinical practice in China and its advantages for non-invasive tumor screening. However, it is important to note that some guidelines^16,17^ prioritize MRI for the staging of rectal cancer, and the choice of modality may present a limitation to our study. Additionally, in clinical applications, an additional classification network, trained using public datasets like TotalSegmentator^58^, is required to assist in the slice selection process. This adds extra time and complicates the auto-triggering of the model. We also devised an automatic point-generation method to incorporate point-based prompts in the evaluation (Supplementary Note 7). However, in real-world evaluations, we observed that the quality of the points and the prompt generation method may impact the model’s performance. Furthermore, prompt annotation time varies significantly depending on user habits and model application. For the dataset, although the ground truth data was rigorously curated, subjective bias from individual clinicians may still persist. The experimental results only suggest that the deep learning model aligns more consistently with the consensus of experts. Additionally, due to the significant workload, the entire intestinal region was not annotated, which may limit its clinical applicability. To ensure more stable evaluation results, we divided the dataset into training and test sets only, and reported the accuracy on the test set.

## Conclusion

In this paper, we construct the first large-scale CT rectal cancer dataset CARE with pixel-level annotations for both normal and cancerous rectum, effectively addressing gaps within the realm of rectal cancer segmentation. This new source of rectal cancer dataset will soon be made publicly available. Inspired by the success of SAM’s innovative promptable (e.g., points) segmentation paradigm, we develop a model U-SAM to achieve better rectal cancer segmentation. The U-SAM model adopts a U-shaped adapter architecture, rectifying the inherent structural limitations of SAM when applied to medical image analysis. Extensive experiments demonstrate that the proposed U-SAM outperforms state-of-the-art methods on CARE and WORD datasets. Through the observer study, the clinical value of our proposed U-SAM was demonstrated, which significantly improves the efficiency and accuracy of rectal cancer diagnosis in clinical practice. In the future, we will still work on extending the CARE dataset to be more extensive and further explore the potential of U-SAM within the domain of medical image segmentation.

## Supplementary information

Supplementary Information

Description of Additional Supplementary Files

Supplementary data 1

Supplementary data 2

Supplementary data 3

Supplementary data 4

Supplementary data 5

Supplementary data 6

Supplementary data 7

Supplementary data 8

Reporting Summary

## Data Availability

The data from The First Affiliated Hospital of Anhui Medical University for CARE dataset used in this study are available at https://github.com/kanydao/U-SAM^59^ to users who agree with our data license (CC BY-NC 4.0) and code license (Apache-2.0 License). The WORD dataset^24^ is available online at https://github.com/HiLab-git/WORD. The source data for Fig. 5 can be found in Supplementary Data 8.

## Code availability

Code for this study is available at https://github.com/kanydao/U-SAM^59^.
