# Supplementary material for: Tuning vision foundation models for rectal cancer segmentation from CT scans
Source: Commun Med (Lond). 2025 Jul 1;5:256. doi: 10.1038/s43856-025-00953-0 (PMC12219254; doi:10.1038/s43856-025-00953-0)
Supplement: Supplementary file 1 — Supplementary Information [file 43856_2025_953_MOESM1_ESM.pdf]

Tuning Vision Foundation Models for Rectal Cancer Segmentation from CT Scans

Hantao Zhang[a,b], Weidong Guo[a,b], Shouhong Wan[a,b,*], Bingbing Zou[b,c,e], Wanqin Wang[b,d,e], Chenyang Qiu[b,c,e], Kaige Liu[b,c,e], Peiquan Jin[a] and Jiancheng Yang[f]

[a] *School of Computer Science and Technology, University of Science and Technology of China, Hefei, China*
[b] *Institute of Artificial Intelligence, Hefei Comprehensive National Science Center, Hefei, China*
[c] *Department of General Surgery, The First Affiliated Hospital of Anhui Medical University, Hefei, China*
[d] *Department of Radiology, The First Affiliated Hospital of Anhui Medical University, Hefei, China*
[e] *Anhui Medical University, Hefei, China*
[f] *Computer Vision Laboratory, Swiss Federal Institute of Technology Lausanne (EPFL), Lausanne, Switzerland*

Supplementary information

Supplementary Note 1. Visualization

In this section, we present a comprehensive discussion of the characteristics of samples within the CARE dataset. Given the intricate anatomical structures of the rectum and the diverse nature of rectal cancer progression, working with the rectal cancer dataset (CARE) poses an exceptionally high level of complexity. Supplementary Fig. 1 illustrates varying sizes and shapes of rectal anatomy in axial cross-sections within the CARE dataset.

The size and shape differ significantly across cases and frames. According to the size, we divide them into five categories. Furthermore, the extent of deterioration in different patients varies, and their anatomical features exhibit substantial differences in morphological characteristics. Supplementary Fig. 2 showcases morphological examples of rectal cancer tumors at various degrees, ranging from normal rectal walls to severe rectal cancer.

Segmentation for irregular anatomical structure: Supplementary Fig. 3 displays the segmentation results of different models in the condition of irregular anatomical structures. As shown in the sixth column of the Supplementary Fig. 3, our proposed U-SAM can better adapt to such complex shape variations and strive to preserve the integrity and completeness of the segmentation label outlines as much as possible.

Supplementary Note 2. Interactive Medical Image Segmentation

Existing large-scale medical segmentation datasets, such as WORD[1] and AMOS[2], primarily focus on annotating organs, which are relatively low-cost and accessible. However, there is a notable scarcity of datasets specifically annotated for cancer tumors, particularly for the rectum, which presents complex anatomical challenges. Mainstream medical image segmentation methods (e.g., TransUnet[3], SwinUnet[4], nnUNet[5]) have shown promising results in segmenting larger organs. However, cancer tumors often exhibit extremely complex morphological characteristics that differ significantly from the structures of larger organs. Moreover, CT images may suffer from low contrast, which can be particularly challenging for segmenting complex structures like cancer tumors and lymph nodes. Conventional medical image segmentation methods often struggle to accurately locate tumor regions and may erroneously segment normal organs as tumors. In our view, medical image segmentation tasks can be divided into two implicit stages. The first stage involves localizing the respective organs and tumors, while the second stage focuses on predicting pixel-level segmentation results for the localized areas. Providing informative cues during the first stage is crucial for tumor segmentation, and previous studies[6] have introduced interactive models based on convolutional neural networks (CNNs) for tumor segmentation. Our proposed U-SAM leveraging the pretrained SAM[7] model, which possesses powerful generalization abilities to address the challenging task of tumor segmentation.

Supplementary Note 3. Trade-offs for Different Prompts

This section offers a comprehensive analysis of different prompt forms in practical medical clinical applications.

The original SAM model supports two independent forms of prompt information: bounding boxes and anchor points. However, not every prompt proves suitable in real-world clinical medical imaging applications. While earlier research[8,9] has indicated that box-based prompts tend to deliver more favorable outcomes than point-based prompts in certain specific scenarios, particularly for segmenting large organs, we opt for point-based prompts for cancer tumor segmentation due to the following reasons.

a. Point-based prompts are more flexible. As illustrated in the first line of Supplementary Fig. 4, certain anatomical structures, including tumors and organs, can

*Corresponding author
wansh@ustc.edu.cn (S. Wan)
ORCID(s): 0009-0002-4368-8223 (S. Wan)

exhibit non-contiguous characteristics within axial cross-sections. For instance, when examining the axial cross-section of the rectal wall (highlighted in red), it is comprised of multiple distinct regions, rendering it unfeasible to encompass them within a single bounding box. Conversely, the use of multiple anchor points as prompts proves to be a more effective approach in representing these disconnected regions. Additionally, as depicted in the second line of Supplementary Fig. 4, employing box-based prompts for two dissimilar anatomical structures, such as tumors and organs, may result in significant overlap. In such scenarios, the inherent imprecision of bounding box prompts exacerbates the ambiguity of the provided prompt information. The spatial prompt information for the rectal wall and rectal tumor is closely intertwined. The larger prompt box (representing the rectum) frequently encompasses another category of anatomical tissues (tumors). This issue can impede the model's ability to distinguish between distinct objects. However, point-based prompts circumvent the problem of ambiguous prompts.

b. Point-based prompts can better represent the morphological characteristics of anatomical structures. The tissues and organs within the human body typically exhibit intricate anatomical complexity. While box-based prompts can offer positional information to some extent, they may fall short in capturing the morphological nuances of complex anatomical structures in some instances. Similar issues have been observed in the MedSAM framework as well[8]. The use of box-based prompts often fails to delineate tiny and low-contrast objects enclosed within the bounding box, as observed in the context of liver tumor segmentation in abdominal CT images. We attribute this issue to the inherent limitations of box-based prompts. Illustrated in Supplementary Supplementary Fig. 5, the rectal wall (depicted in red) is a hollow anatomical structure that may contain tumors and impurities. The direct utilization of box-level prompt information may inadvertently introduce irrelevant organizational details. When multiple similar instances, such as tumors and impurities, surround the target for segmentation, relying solely on bounding boxes can result in inaccurate segmentation outcomes. Alternatively, point-based prompts can efficiently outline the contours of the rectal wall at a relatively lower computational cost, as demonstrated by the yellow points in Supplementary Fig. 5.

c. Point-based prompts are more generalized. SAM[7] is initially designed for binary segmentation task: segmenting the elements of foreground and background, respectively. For every image, it can only accept the maximum of one box prompt for the foreground and multiple anchor points for the foreground or background. Recently, some studies[8,10] have showcased the effectiveness of using box-based prompts, particularly for organ segmentation within medical imaging scenarios. In this paradigm, each category of objects' result requires inference independently at least once to obtain the final results. However, box-based prompts encounter challenges when extending to the full-category segmentation tasks in certain medical domains. For SAM's promptable segmentation paradigm, we define full-category segmentation tasks as follows: the interactive model receives the prompts for all category-specific objects at the beginning and generates segmentation masks for all category-specific objects in a single inference process. Thus, when building the model based on the box-based prompt, it can only accept the ROI (region of interest)prompt information rather than the category information.

In cases where the dataset contains an extensive array of categories, SAM's limitation of accepting only one box per inference results in a generally large prompt box. As a consequence, the ROI tends to be relatively indistinct. In the main paper, experiments were conducted on the CARE and WORD datasets to assess the performance of box-based prompts. On the CARE dataset, box-based prompts exhibited slightly better performance than point-based methods. This improvement may be attributed to the CARE dataset's focus on the rectal area, where the region of interest is relatively small. Moreover, box-based prompts can offer relatively accurate ROI information. Conversely, on the WORD dataset, box-based prompts performed even worse than the model without any prompt information. It's important to note that the WORD dataset owns a larger number of categories and often contains multiple categories of objects within a single image that need to be segmented concurrently. When the model employs box-based prompts, the box typically encompasses the entire image, making it challenging to provide valuable spatial information. In contrast, point-based prompts are not encumbered by this issue. Multiple category-agnostic anchor point prompts can simultaneously convey location and category information. Our proposed U-SAM can fully leverage these point-based prompts to perform full-category segmentation on input images in a single inference, circumventing the limitations of box-based prompts.

Supplementary Note 4. Generalization on WORD

Performance: Supplementary Table 2 showcases the performance of our proposed U-SAM on the WORD dataset. All the SAM-based models utilized 'ViT-B', and the experiments yielded conclusions similar to those on the CARE dataset. However, we observed that for the WORD dataset, point prompts provided better segmentation results compared to box prompts.

Computational Cost: Supplementary Fig. 6 illustrates the comparison of performance and computational cost for various state-of-the-art SAM-based medical segmentation methods on the WORD dataset. The figure demonstrates that our proposed U-SAM+LoRA achieves an excellent balance between computational cost and performance. Supplementary Table 3 provides a detailed comparison of performance variations across different state-of-the-art medical segmentation methods on the WORD dataset.

Supplementary Note 5. Different Model Variations

In this section, we present a more detailed analysis of the performance of various model variants on the CARE and WORD datasets. As illustrated in Supplementary Table 5, our proposed U-SAM outperforms the original SAM in both normal rectal wall and tumor segmentation across different model scales. This improved performance can be attributed to the design of the convolution module and the integration of skip connections. Similar trends are observed in the Supplementary Table. 3 for the WORD dataset. U-SAM consistently delivers competitive segmentation results for a wide range of organ structures in the WORD dataset. Concurrently, we observed similar experimental results to previous studies [11]. SAM's lightweight ViT-B model exhibits robust competitiveness on both datasets, suggesting its suitability for transfer to medical image applications.

Supplementary Note 6. Annotation Details

Supplementary Fig. 13 depicts the annotation tools employed in our work. We utilize the ITK-SNAP annotation tools [12] to meticulously annotate pixel-level details for both normal and cancerous rectum regions. The initial phase involves collaborative analysis by a gastrointestinal surgery clinician and a radiologist, focusing on the patient's medical condition and the precise location of rectal cancer. Following this, the labeling process is initiated under the guidance of clinical expertise, conducted slice by slice. This labeling procedure is structured into two distinct stages. In the first round, medical professionals outline the general boundaries of the normal rectum and the tumor.

In the second round, the edge burrs and discontinuous parts are corrected with the three-dimensional stereoscopic views. This meticulous two-step labeling approach ensures the utmost accuracy of the datasets. Since the original CT data encompasses the entire body, we have taken strategic measures to enhance training efficiency by eliminating extraneous regions. Slices lacking the presence of the rectum are systematically excluded, and the corresponding images and labels are compactly organized into pairs. These pairs are then stored in the npz format native to Python. Labeled case samples are provided in the accompanying folder.

Supplementary Note 7. Prompt Generation

Suppose that all the prompts supplied to our model originate from clinical diagnosis professionals. Consequently, the promotable points utilized within the U-SAM framework are derived from posterior label knowledge. Under this premise, we developed a novel prompt sampling strategy. Specifically, for each object class, a predetermined quantity of points is sampled (e.g., 3 points per class) to create prompts. Drawing inspiration from the methodology presented in SAM [7], we employ a stochastic sampling strategy during the training phase to enhance the model's adaptability to diverse prompts. Conversely, when dealing with the testing dataset, a deterministic strategy is employed to ensure the uniformity of the evaluation process. The visual representation of the automatically generated qualitative prompts for input samples is depicted in Supplementary Fig. 12, wherein the yellow dots symbolize the points selected from the ground-truth annotations.

Supplementary Note 8. Implementation Details

This section offers a more comprehensive exposition of the configuration of our proposed U-SAM. Further details can be found in the accompanying code folder.

Loss Function Following the previous works, we adopt a loss function consisting of the linear combination of Dice loss and cross entropy loss. The loss function can be formulated as follows:

$$\mathcal{L} = \alpha\mathcal{L}_{Dice} + (1 - \alpha)\mathcal{L}_{CE} \tag{1}$$

where $\mathcal{L}_{Dice}$ and $\mathcal{L}_{CE}$ denotes Dice loss and cross entropy loss, respectively. Regarding the coefficient in the loss function, we judiciously set α to 0.6, encouraging the model to prioritize both intricate target contours and the accurate identification of their corresponding classes.

Training Strategy To effectively align with the new U-SAM architecture, we implement a corresponding training strategy that enhances the convergence rate. Specifically, we begin by importing pre-trained SAM weights into our U-SAM, bolstering training stability and accelerating model convergence. Distinct learning rates are applied to different components of our model, customized to their respective roles and contributions within the architecture. Specifically, the learning rate for the vision transformer is set to a relatively small value of 10^{-4} to preserve and leverage its knowledge acquired from the natural domain. For the remaining trainable components of U-SAM (i.e., the convolutional encoder and decoder), we carefully select a learning rate of 10^{-3} to facilitate their faster adaptation to the medical domain. In particular, we assign a deliberately small learning rate of 10^{-4} to the vision transformer to preserve and leverage the knowledge gained from the natural domain. This thoughtful differentiation allows for a balanced adaptation, where the convolutional encoding and decoding modules, along with the mask decoder, are set to 10^{-3} to accelerate their alignment to the medical context.

Observer Study Details: The clinical test dataset includes 20 unique patient cases, resulting in a total of 297 annotated cases. We ensured that key characteristics of this subset, such as T stage, N stage, and gender distribution, were consistent with the larger dataset to maintain representativeness. Specific parameters are detailed in Supplementary Table 4.

Supplementary Note 9. 3D Segmentation

As a tubular organ, the rectum exhibits considerable variation in size and shape across different frames, presenting

unique characteristics compared to typical organs in three-dimensional space. Furthermore, our study not only focuses on segmenting the normal rectum, as previous work [13] has done, but also addresses rectal tumors. The complexity of tumor identification and the diversity of tumor morphology further exacerbate the challenges associated with their segmentation. Additionally, the relatively small dataset for rectal cancer, compared to other organs, is insufficient to support the training of a robust 3D model. In practice, we observed that the performance of 3D segmentation was even lower than that of 2D segmentation.

Supplementary Table 6 reports the segmentation performance of the 3D U-Net [14] model across different regions (Normal and Tumor) and the overall mean. The evaluation metrics include Dice coefficient, Intersection over Union (IoU), and Normalized Surface Dice (NSD). We trained the 3D U-Net [14] using a learning rate of 1×10^{-5} and the Adam optimizer to ensure smooth convergence of the model. As shown in Supplementary Table 6, the overall mean performance was 52.88% Dice, 37.51% IoU, and 26.66% NSD, all of which are lower than those of the 2D model discussed in the main text.

We further visualizes the segmentation results of the 3D U-Net in Supplementary Fig. 14. The first and third rows display the ground truth labels for the normal rectum and rectal tumor, respectively, while the second and fourth rows show the segmentation results produced by the 3D model.As illustrated in columns 1, 2, and 3 of Supplementary Fig. 14, the 3D implementation struggles to distinguish between the normal rectum and the tumor, often resulting in large, continuous misclassifications. Additionally, when the tumor morphology is more complex, the 3D segmentation frequently fails to accurately capture the corresponding 3D morphological features, as demonstrated in columns 4 and 5.

Supplementary Note 10. Potential Research Topics

This section offers an introductory glimpse into forthcoming clinical application research possibilities that can be explored using our extensive and meticulously annotated pixel-level CT image dataset of rectal cancer. It is worth noting that the rectum typically occupies a relatively small portion of the entire body in a CT scan. Consequently, this aspect introduces additional complexities and challenges to the corresponding diagnostic processes.

Our proposed CARE dataset thoroughly captures the most authentic scenarios encountered in clinical practice and can be used to develop or evaluate clinical applications. Similar to other comprehensive datasets [1,15] in the medical domain, it can also be extended for use in broader algorithmic research domains, encompassing fully-/semi-/weakly-supervised learning, domain adaptation/generalization, partial labeling, and more.

Supplementary References

[1] X. Luo, W. Liao, J. Xiao, J. Chen, T. Song, X. Zhang, K. Li, D. N. Metaxas, G. Wang, S. Zhang, Word: A large scale dataset, benchmark and clinical applicable study for abdominal organ segmentation from ct image, Medical Image Analysis 82 (2022) 102642.

[2] Y. Ji, H. Bai, C. Ge, J. Yang, Y. Zhu, R. Zhang, Z. Li, L. Zhanng, W. Ma, X. Wan, et al., Amos: A large-scale abdominal multi-organ benchmark for versatile medical image segmentation, Advances in Neural Information Processing Systems 35 (2022) 36722–36732.

[3] J. Chen, Y. Lu, Q. Yu, X. Luo, E. Adeli, Y. Wang, L. Lu, A. L. Yuille, Y. Zhou, Transunet: Transformers make strong encoders for medical image segmentation, arXiv preprint arXiv:2102.04306 (2021).

[4] H. Cao, Y. Wang, J. Chen, D. Jiang, X. Zhang, Q. Tian, M. Wang, Swin-unet: Unet-like pure transformer for medical image segmentation, in: European conference on computer vision, Springer, 2022, pp. 205–218.

[5] F. Isensee, P. F. Jaeger, S. A. Kohl, J. Petersen, K. H. Maier-Hein, nnu-net: a self-configuring method for deep learning-based biomedical image segmentation, Nature methods 18 (2021) 203–211.

[6] T. Zhou, L. Li, G. Bredell, J. Li, J. Unkelbach, E. Konukoglu, Volumetric memory network for interactive medical image segmentation, Medical Image Analysis 83 (2023) 102599.

[7] A. Kirillov, E. Mintun, N. Ravi, H. Mao, C. Rolland, L. Gustafson, T. Xiao, S. Whitehead, A. C. Berg, W.-Y. Lo, et al., Segment anything, arXiv preprint arXiv:2304.02643 (2023).

[8] J. Ma, Y. He, F. Li, L. Han, C. You, B. Wang, Segment anything in medical images, Nature Communications 15 (2024) 654.

[9] J. Wu, R. Fu, H. Fang, Y. Liu, Z. Wang, Y. Xu, Y. Jin, T. Arbel, Medical sam adapter: Adapting segment anything model for medical image segmentation, arXiv preprint arXiv:2304.12620 (2023).

[10] K. Zhang, D. Liu, Customized segment anything model for medical image segmentation, arXiv preprint arXiv:2304.13785 (2023).

[11] Y. Li, M. Hu, X. Yang, Polyp-sam: Transfer sam for polyp segmentation, arXiv preprint arXiv:2305.00293 (2023).

[12] P. A. Yushkevich, J. Piven, H. C. Hazlett, R. G. Smith, S. Ho, J. C. Gee, G. Gerig, User-guided 3d active contour segmentation of anatomical structures: significantly improved efficiency and reliability, Neuroimage 31 (2006) 1116–1128.

[13] M. Lempart, M. P. Nilsson, J. Scherman, C. J. Gustafsson, M. Nilsson, S. Alkner, J. Engleson, G. Adrian, P. Munck af Rosenschöld, L. E. Olsson, Pelvic u-net: multi-label semantic segmentation of pelvic organs at risk for radiation therapy anal cancer patients using a deeply supervised shuffle attention convolutional neural network, Radiation Oncology 17 (2022) 114.

[14] Ö. Çiçek, A. Abdulkadir, S. S. Lienkamp, T. Brox, O. Ronneberger, 3d u-net: learning dense volumetric segmentation from sparse annotation, in: Medical Image Computing and Computer-Assisted Intervention–MICCAI 2016: 19th International Conference, Athens, Greece, October 17-21, 2016, Proceedings, Part II 19, Springer, 2016, pp. 424–432.

[15] S. Gatidis, T. Hepp, M. Früh, C. La Fougère, K. Nikolaou, C. Pfannenberg, B. Schölkopf, T. Küstner, C. Cyran, D. Rubin, A whole-body fdg-pet/ct dataset with manually annotated tumor lesions, Scientific Data 9 (2022) 601.

[16] E. J. Hu, P. Wallis, Z. Allen-Zhu, Y. Li, S. Wang, L. Wang, W. Chen, et al., Lora: Low-rank adaptation of large language models, in: International Conference on Learning Representations, 2021.

[17] M. Naderi, M. Givkashi, F. Piri, N. Karimi, S. Samavi, Focal-unet: Unet-like focal modulation for medical image segmentation, arXiv preprint arXiv:2212.09263 (2022).

[18] M. Z. Alom, C. Yakopcic, M. Hasan, T. M. Taha, V. K. Asari, Recurrent residual u-net for medical image segmentation, Journal of Medical Imaging 6 (2019) 014006–014006.

[19] D. Jha, P. H. Smedsrud, M. A. Riegler, D. Johansen, T. De Lange, P. Halvorsen, H. D. Johansen, Resunet++: An advanced architecture for medical image segmentation, in: 2019 IEEE international symposium on multimedia (ISM), IEEE, 2019, pp. 225–2255.

[20] N. Ibtehaz, M. S. Rahman, Multiresunet: Rethinking the u-net architecture for multimodal biomedical image segmentation, Neural networks 121 (2020) 74–87.

[21] X. Huang, Z. Deng, D. Li, X. Yuan, Y. Fu, Missformer: An effective transformer for 2d medical image segmentation, IEEE Transactions on Medical Imaging (2022).

[22] H. Wang, P. Cao, J. Wang, O. R. Zaiane, Uctransnet: rethinking the skip connections in u-net from a channel-wise perspective with transformer, in: Proceedings of the AAAI conference on artificial intelligence, volume 36, 2022, pp. 2441–2449.

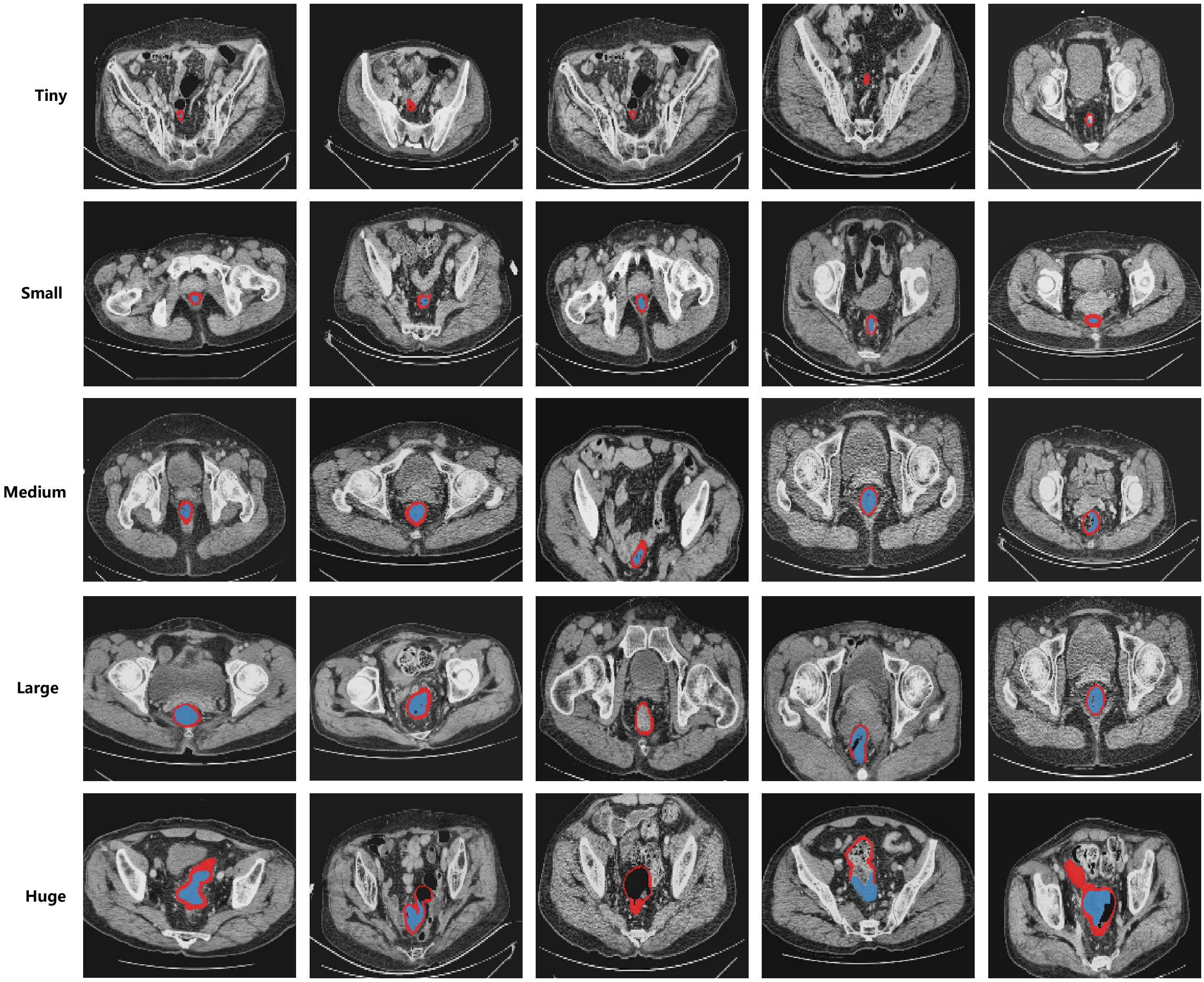

Supplementary Figure 1: Visualization of different sizes and shapes of rectal wall and cancer tumors in the CARE dataset. Red indicates normal rectal tissue, while the blue represents rectal cancer tumors.

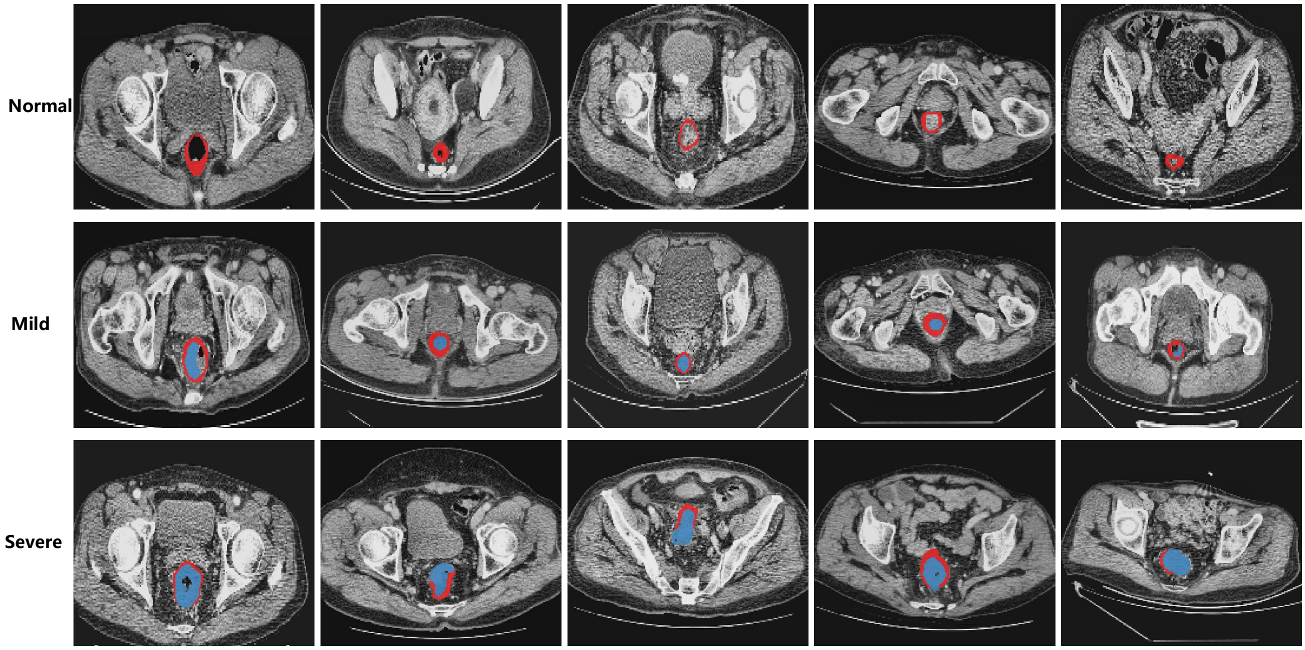

Supplementary Figure 2: Visualization of different levels of rectal cancer tumor deterioration in the CARE dataset. Red indicates normal rectal tissue, while the blue represents rectal cancer tumors.

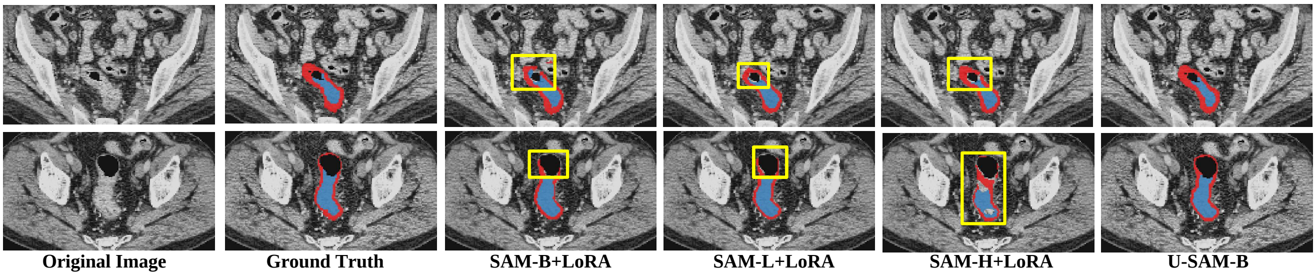

Supplementary Figure 3: The qualitative comparison of irregular anatomy segmentation results on the CARE dataset. Red indicates normal rectal tissue, while the blue represents rectal cancer tumors.

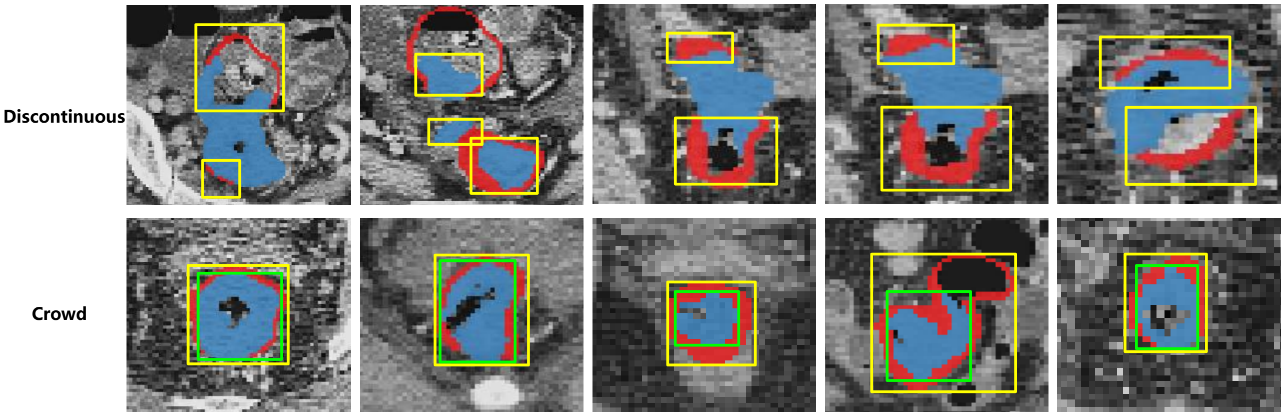

Supplementary Figure 4: Some challenging case for box-based prompts in the CARE dataset. Red indicates normal rectal tissue, while the blue represents rectal cancer tumors.

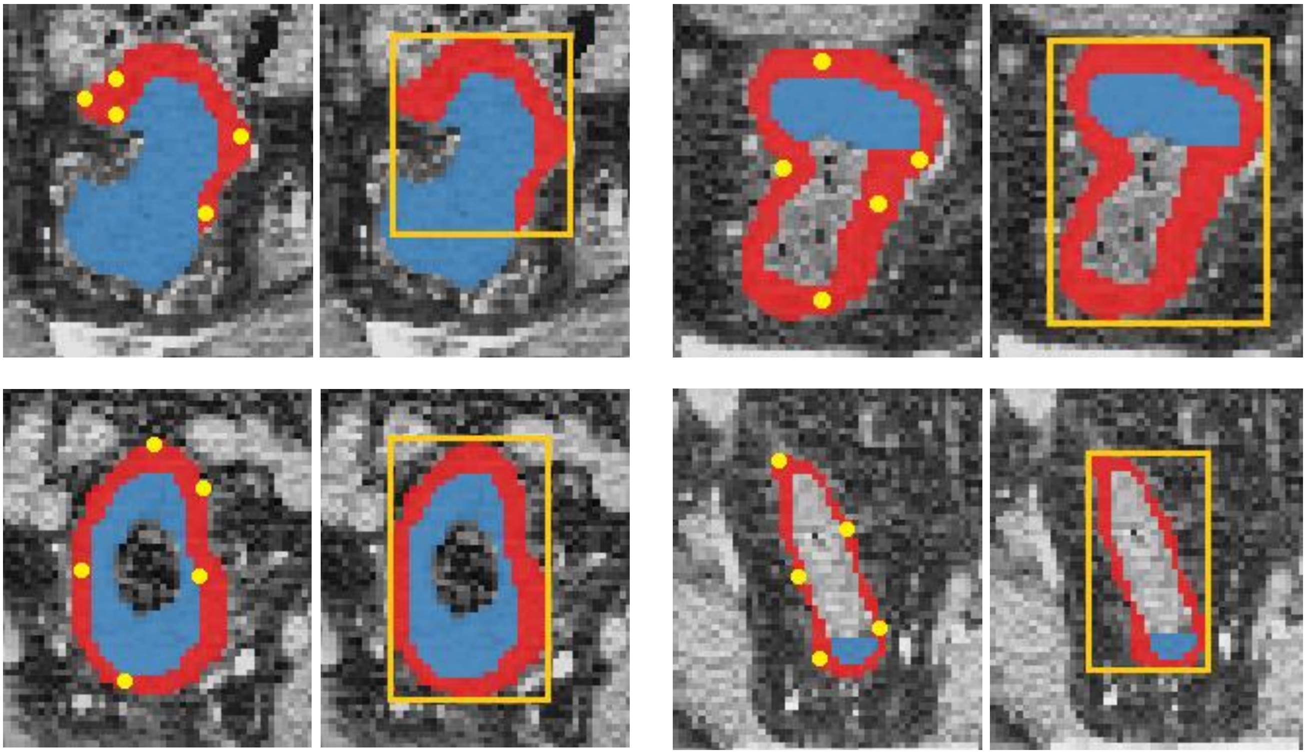

Supplementary Figure 5: Comparison of morphological characteristics represented by point-based prompts and box-based prompts. Red indicates normal rectal tissue, while the blue represents rectal cancer tumors.

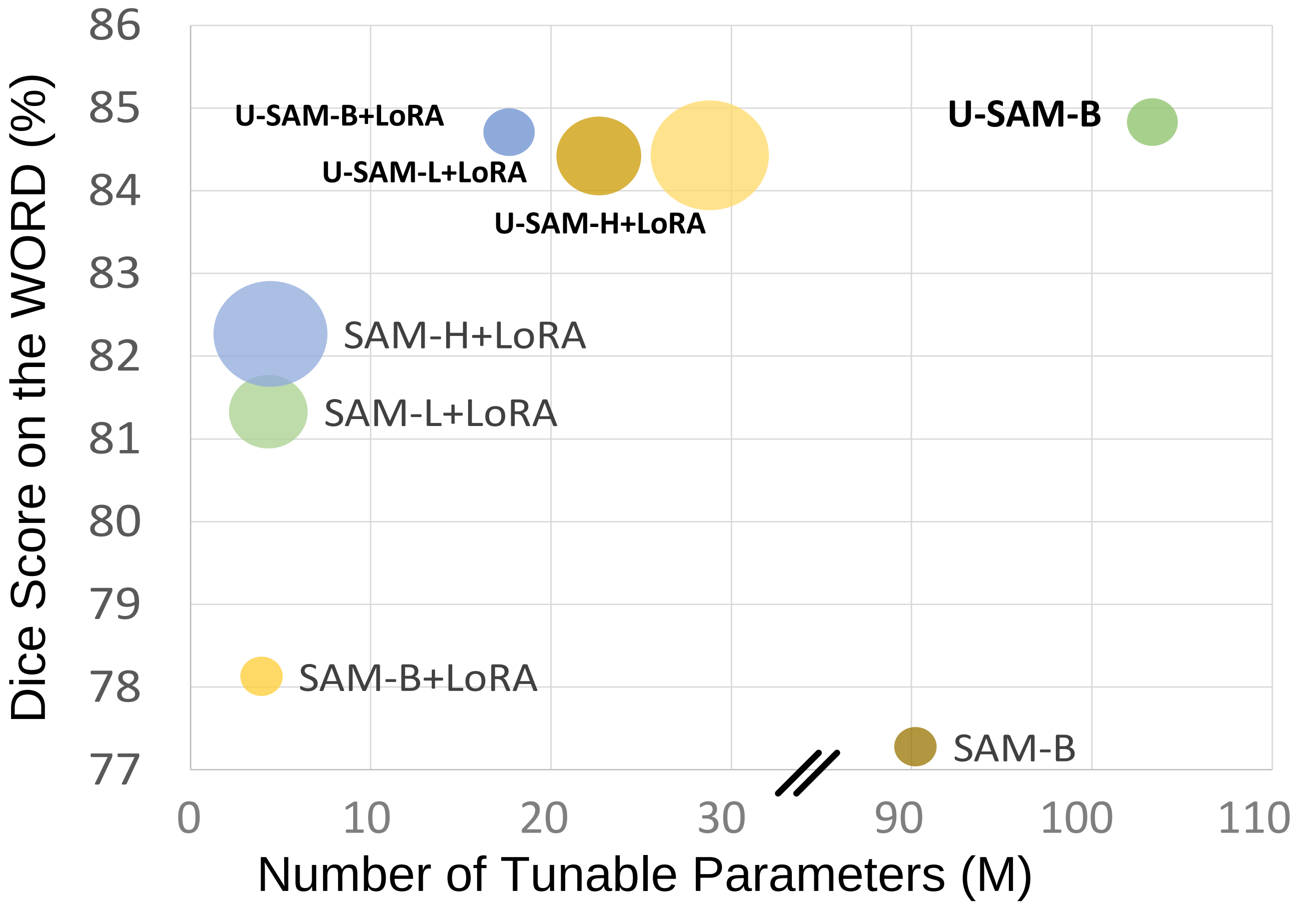

Supplementary Figure 6: Performance comparison of state-of-the-art SAM-based medical segmentation methods on the WORD dataset. The bubble size represents GFLOPS. 'LoRA' denotes the model implemented using Low-Rank Adaptation [16].

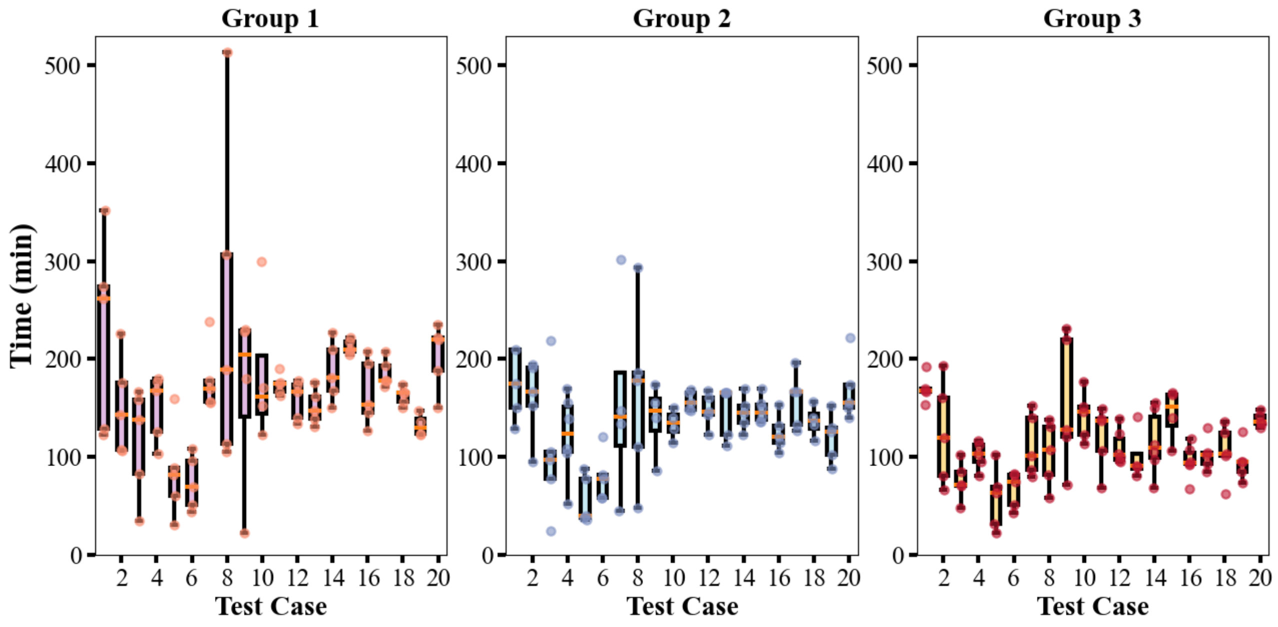

Supplementary Figure 7: Statistical analysis of annotation time by clinicians. For twenty clinical trial test cases, each case was independently annotated by three groups of clinicians, categorized as Group 1, Group 2, and Group 3 (from left to right). Each clinician's annotation time is presented as a scatter plot on the graph, with a box plot for each category included. Each box extends from the lower to upper quartile values of the annotation times within the same group of clinicians, with a line at the median.

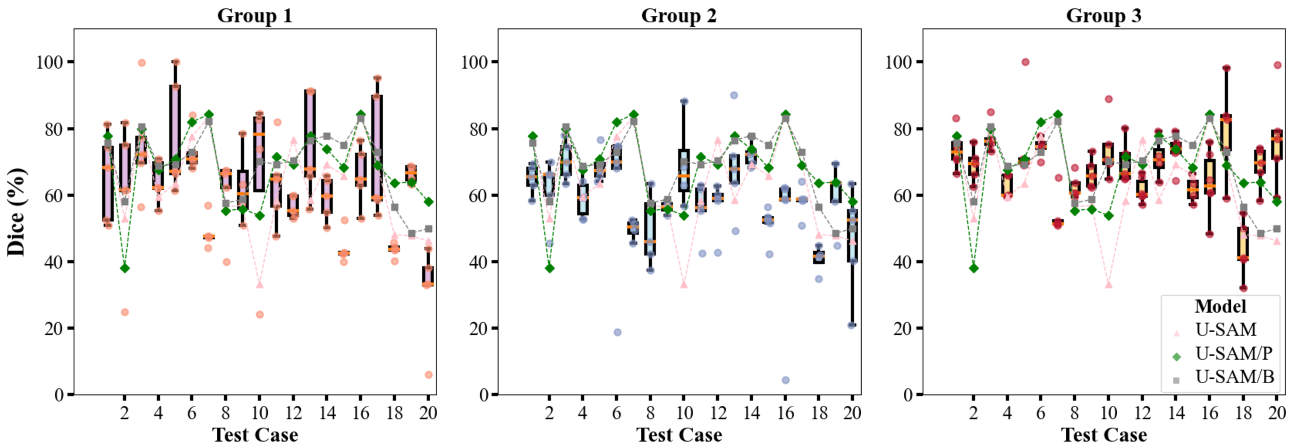

Supplementary Figure 8: Statistical analysis of segmentation diagnostic accuracy by clinicians. For twenty clinical trial test cases, each case was independently annotated by three groups of clinicians, categorized as Group 1, Group 2, and Group 3 (from left to right). Each clinician's annotations were compared with the ground truth, and the Dice coefficient values were calculated to assess segmentation accuracy. Each box extends from the lower to the upper quartile values of the annotation times within the same group of clinicians, with a line at the median. The Dice scores of our different variant models are also plotted on the graph as dashed lines for reference.

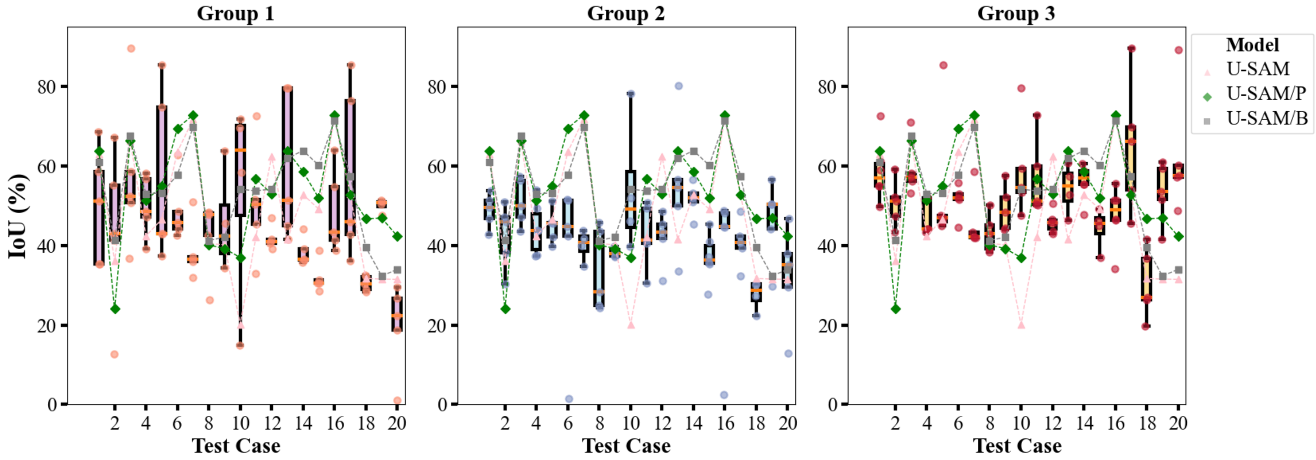

Supplementary Figure 9: Statistical analysis of segmentation diagnostic accuracy by clinicians. For twenty clinical trial test cases, each case was independently annotated by three groups of clinicians, categorized as Group 1, Group 2, and Group 3 (from left to right). Each clinician's annotations were compared with the ground truth, and the IoU were calculated to assess segmentation accuracy. Each box extends from the lower to the upper quartile values of the annotation times within the same group of clinicians, with a line at the median. The IoU scores of our different variant models are also plotted on the graph as dashed lines for reference.

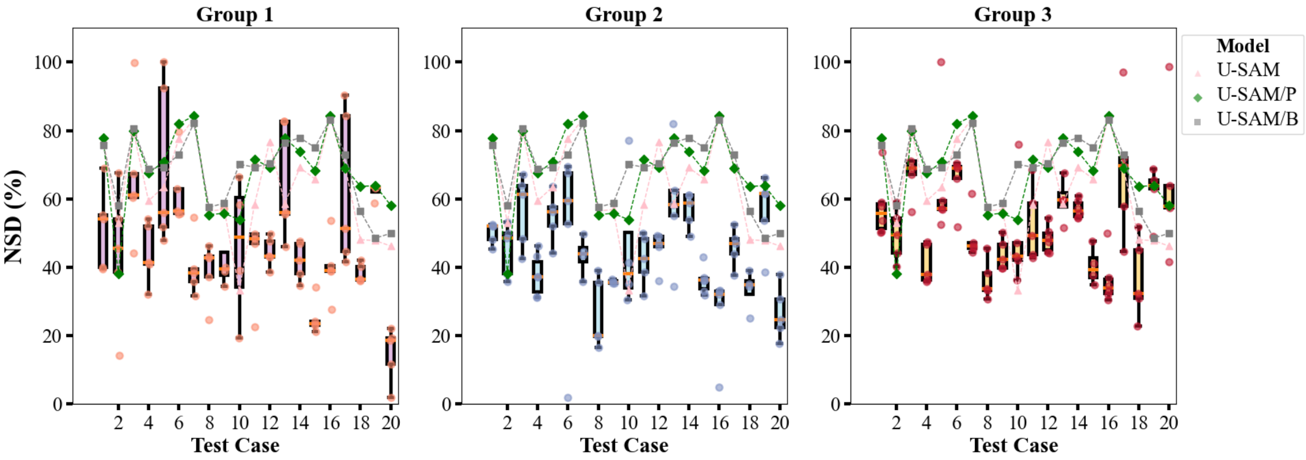

Supplementary Figure 10: Statistical analysis of segmentation diagnostic accuracy by clinicians. For ten clinical trial test cases, each case was independently annotated by three groups of clinicians, categorized as Group 1, Group 2, and Group 3 (from left to right). Each clinician's annotations were compared with the ground truth, and the NSD were calculated to assess segmentation accuracy. Each box extends from the lower to the upper quartile values of the annotation times within the same group of clinicians, with a line at the median. The NSD scores of our different variant models are also plotted on the graph as dashed lines for reference.

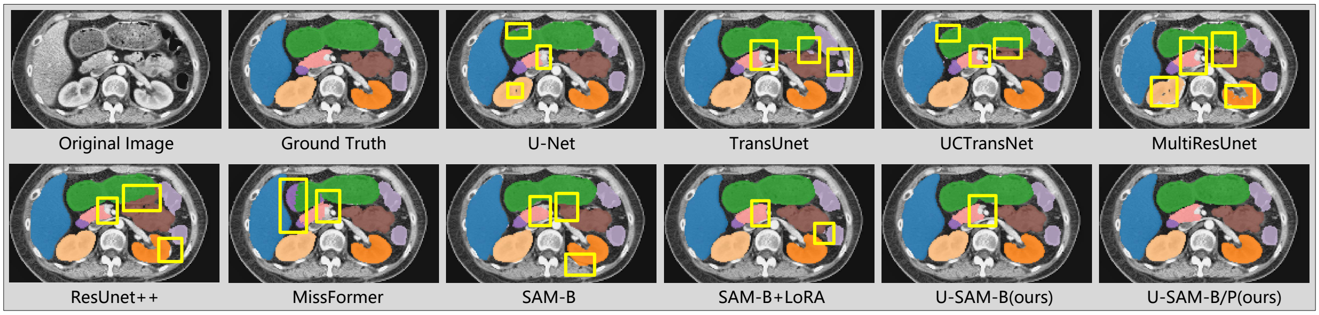

Supplementary Figure 11: The qualitative comparison on the WORD dataset.

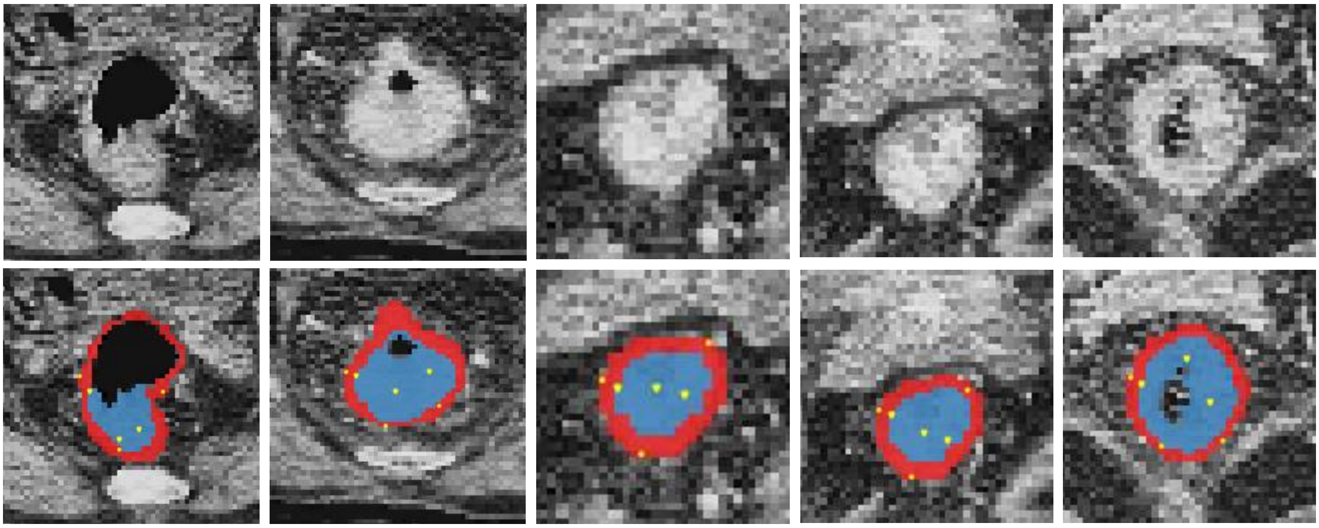

Supplementary Figure 12: The example of the automatically generated points prompt. Red indicates normal rectal tissue, while the blue represents rectal cancer tumors.

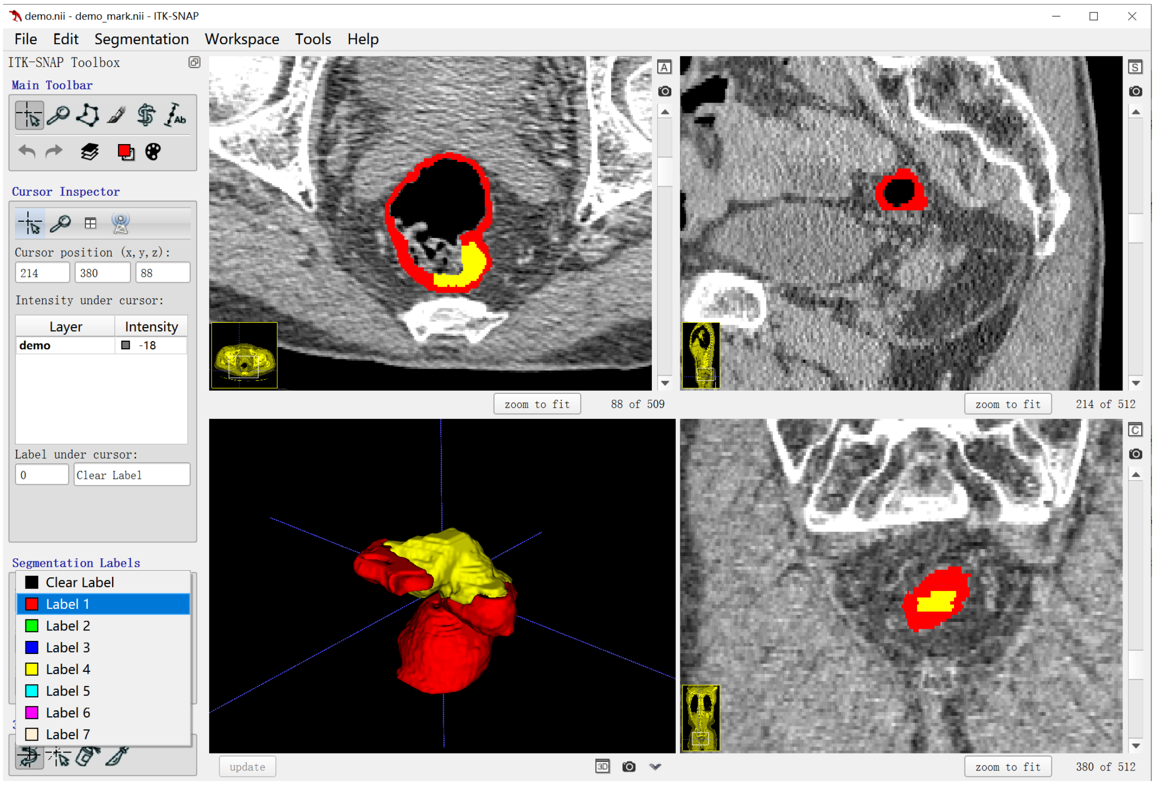

Supplementary Figure 13: ITK-SNAP: the annotation tool employed for annotating CARE.

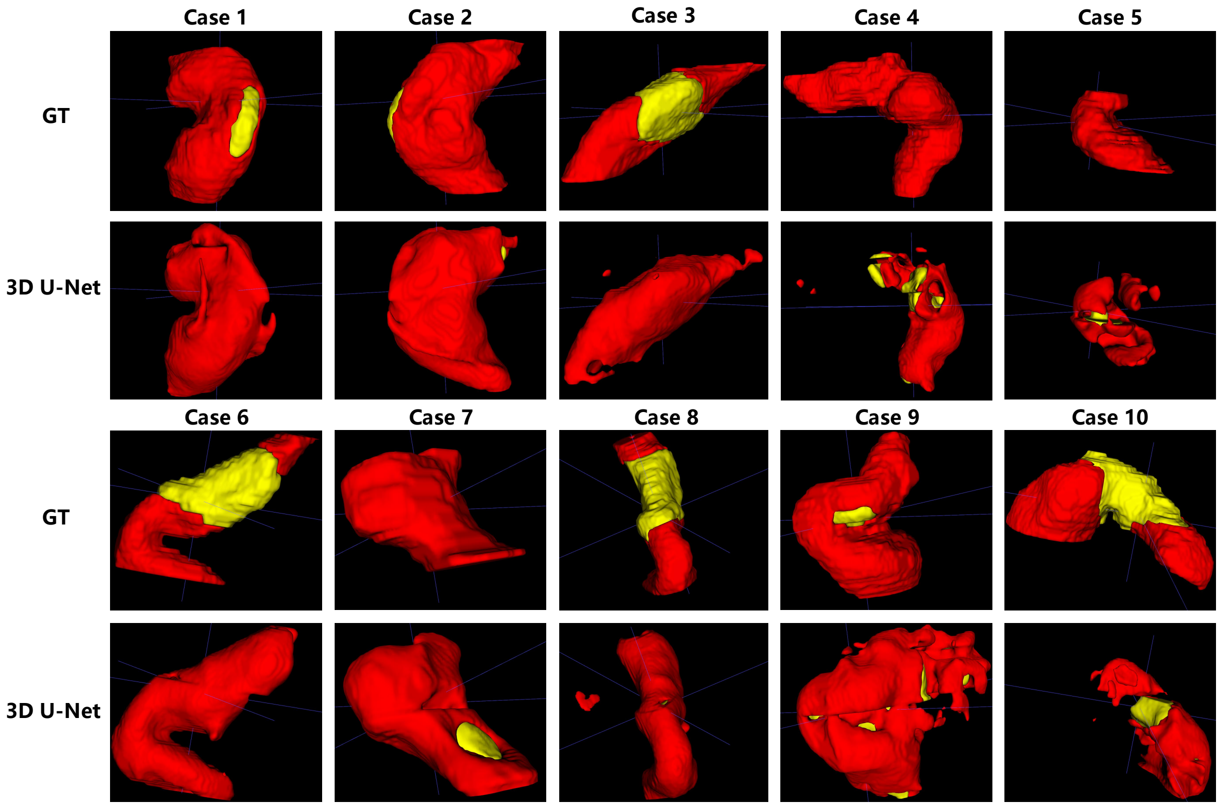

Supplementary Figure 14: Visualization of 3D U-Net[14] Segmentation Results. 'GT' indicates the ground truth. Red represents normal rectal tissue, while yellow indicates rectal cancer tumors.

	Total	Training (80%)	Test (20%)
**Gender**			
Male	258	205 (80%)	53 (20%)
Female	140	112 (80%)	28 (20%)
**Age (mean±sd years)**	63.01±11.51	63.12±11.62	62.60±11.15
**Tumor Size (mean±sd cm)**	5.88±3.05	5.76±2.21	6.33±5.17
**Tumor Location**			
Low	149	117 (79%)	32 (21%)
Medium	200	156 (78%)	44 (22%)
High	49	44 (90%)	5 (10%)
**PIN**			
Negative	245	196 (80%)	49 (20%)
Positive	153	121 (79%)	32 (21%)
**Lymphatic invasion**			
Negative	261	209 (80%)	52 (20%)
Positive	137	108 (79%)	29 (21%)
**T Stage (Tumor Stage)**			
T1	25	21 (84%)	4 (16%)
T2	96	75 (78%)	21 (22%)
T3	226	176 (78%)	50 (22%)
T4	51	45 (88%)	6 (12%)
**N Stage (Tumor Stage)**			
N0	198	156 (79%)	42 (21%)
N1	124	100 (81%)	24 (19%)
N2	76	61 (80%)	15 (20%)
**Height (mean±sd cm)**	165.53±6.82	165.34±6.77	166.23±7.01
**Weight (mean±sd kg)**	61.56±9.92	61.90±10.00	60.24±9.53
**BMI (mean±sd kg/m²)**	22.43±3.10	22.59±3.10	21.78±3.05
**AFP**			
Negative (<7 ng/ml)	387	309 (80%)	78 (20%)
Positive (>7 ng/ml)	11	8 (73%)	3 (27%)
**CEA**			
Negative (<5 ng/ml)	274	221 (81%)	53 (19%)
Positive (>5 ng/ml)	124	96 (77%)	28 (23%)
**CA 125**			
Negative (<35 U/ml)	386	309 (80%)	77 (20%)
Positive (>35 U/ml)	12	8 (67%)	4 (33%)
**CA 19-9**			
Negative (<35 U/ml)	361	288 (80%)	73 (20%)
Positive (>35 U/ml)	37	29 (78%)	8 (22%)

Supplementary Table 1

Clinical Baseline Data of the CARE Dataset. Here, 'PIN' represents Prostatic Intraepithelial Neoplasia; 'AFP' stands for Alpha Fetoprotein; 'CEA' denotes Carcinoembryonic Antigen; 'CA 125' and 'CA 19-9' refer to Cancer Antigen 125 and Cancer Antigen 19-9, respectively. 'Tumor size' refers to the maximum length of the tumor's major axis. The number in parentheses represents the proportion of each cohort.

Model	Liver	Spleen	Kidney (L)	Kidney (R)	Stomach	Gallbladder	Esophagus	Pancreas	Duodenum	Colon	Intestine	Adrenal	Rectum	Bladder	HFL	HFR	Mean
FocalUnet [17]	93.21	89.54	88.64	88.68	83.43	61.29	57.83	60.57	45.20	70.72	72.47	48.03	70.08	90.47	84.63	83.77	74.28
R2Unet [18]	84.73	90.35	90.56	87.78	80.21	59.56	71.12	72.64	49.74	70.77	73.30	48.26	72.99	88.20	74.17	47.78	72.63
ResUnet++ [19]	95.08	93.71	93.92	94.22	89.28	69.28	72.95	75.82	57.15	79.80	80.73	65.59	75.27	93.20	**92.26**	**92.01**	82.52
MultiResUnet [20]	95.19	93.73	93.12	93.33	90.73	69.83	73.11	75.33	60.36	81.32	82.51	64.51	78.35	93.57	85.25	87.94	82.30
MissFormer [21]	85.65	94.60	91.00	91.30	90.22	71.62	72.27	76.02	57.85	80.44	80.87	64.02	76.55	93.53	87.26	86.90	81.89
SwinUnet-B [4]	94.91	91.73	89.80	89.76	90.43	70.05	72.33	74.01	56.69	79.85	80.47	61.67	78.01	93.27	87.71	87.82	81.16
SwinUnet-L [4]	95.19	92.69	89.87	89.94	90.45	72.97	72.66	72.89	58.37	79.67	80.51	59.77	77.55	93.63	87.76	87.69	81.37
TransUnet-B [3]	95.46	93.21	91.47	91.63	90.01	70.99	70.61	75.38	55.47	78.73	81.25	64.74	76.66	93.76	87.12	87.56	81.50
TransUnet-L [3]	94.93	89.88	90.56	90.47	91.62	75.52	75.17	76.51	60.41	81.78	83.18	67.33	79.63	94.33	88.40	88.07	82.99
UCTransNet [22]	95.19	94.18	94.27	94.62	89.04	65.83	68.67	73.30	58.44	79.60	80.59	64.36	75.43	92.23	89.31	89.79	81.55
nnUNet [5]	95.44	93.91	94.55	94.60	89.63	66.56	74.78	78.85	63.57	82.45	85.41	65.85	72.42	92.42	84.76	77.58	82.05
SAM-B [7]	94.50	91.67	89.44	88.91	87.77	59.83	61.90	70.15	51.53	71.91	75.83	51.71	72.77	91.91	88.24	88.34	77.28
**U-SAM-B**	95.47	**94.94**	**95.33**	**95.46**	91.66	**76.91**	77.91	75.58	**65.60**	**83.38**	83.27	69.39	80.66	94.20	88.23	88.31	84.83
**U-SAM-B/P**	**96.04**	94.85	92.57	92.68	**91.96**	75.15	**81.51**	**79.18**	65.54	82.73	**83.99**	**73.15**	**82.94**	**95.03**	89.81	89.86	**85.44**
**U-SAM-B/B**	95.87	94.30	92.61	92.73	91.92	76.48	76.84	79.34	64.10	83.29	83.52	68.72	79.74	94.70	89.66	89.75	84.60

Supplementary Table 2

Comparisons of performance with existing methods on the WORD dataset. 'B' denotes that the model utilizes the 'ViT-B', while 'H' indicates the 'ViT-H'. 'U-SAM-B/P' refers to the model incorporating 3 points prompt per class. 'U-SAM-B/B' refers to the U-SAM model utilizing the box-based prompts. HFL:Head of Femur (L), HFR:Head of Femur(R).

Model	Liver	Spleen	Kidney (L)	Kidney (R)	Stomach	Gallbladder	Esophagus	Pancreas	Duodenum	Colon	Intestine	Adrenal	Rectum	Bladder	HFL	HFR	Mean
UCTransNet [22]	95.19	94.18	94.27	94.62	89.04	65.83	68.67	73.30	58.44	79.60	80.59	64.36	75.43	92.23	89.31	89.79	81.55
TransUnet-B [3]	95.46	93.21	91.47	91.63	90.01	70.99	70.61	75.38	55.47	78.73	81.25	64.74	76.66	93.76	87.12	87.56	81.50
SwinUnet-B [4]	94.62	92.72	89.32	89.31	88.85	70.92	66.36	70.11	53.20	78.64	78.46	55.13	76.32	92.34	86.80	86.57	79.35
TransUnet-L [3]	94.93	89.88	90.56	90.47	91.62	75.52	75.17	76.51	60.41	81.78	83.18	67.33	79.63	94.33	88.40	88.07	82.99
SwinUnet-L [4]	95.19	92.69	89.87	89.94	90.45	72.97	72.66	72.89	58.37	79.67	80.51	59.77	77.55	93.63	87.76	87.69	81.37
SAM-B [7]	94.50	91.67	89.44	88.91	87.77	59.83	61.90	70.15	51.53	71.91	75.83	51.71	72.77	91.91	88.24	88.34	77.28
SAM-B+LoRA [10]	94.50	92.61	90.16	90.37	88.01	63.69	65.98	68.95	50.43	75.23	76.42	53.74	74.34	93.09	86.24	86.33	78.13
U-SAM-B	95.47	94.94	95.33	95.46	91.66	76.91	77.91	75.58	65.60	83.38	83.27	69.39	80.66	94.20	88.23	88.31	84.83
U-SAM-B+LoRA	96.06	95.55	93.87	93.99	91.85	73.95	77.64	78.72	64.08	84.00	84.16	69.50	80.60	94.36	88.29	88.66	84.71
SAM-L [7]	94.95	93.21	93.50	93.32	88.45	58.96	62.88	71.91	53.10	73.43	77.29	54.50	74.00	92.67	81.52	81.87	77.85
SAM-L+LoRA [10]	95.31	94.12	90.35	90.33	90.33	71.04	71.31	93.61	58.09	79.49	80.42	58.93	78.88	93.94	87.38	87.75	81.33
U-SAM-L	95.81	95.22	93.37	93.46	91.78	75.89	78.42	79.66	63.12	83.77	84.00	66.19	80.07	94.07	89.73	87.66	84.51
U-SAM-L+LoRA	95.92	94.89	92.49	92.56	91.71	75.23	78.09	78.68	64.11	83.92	84.27	68.59	80.10	94.57	87.61	88.01	84.42
SAM-H [7]	NaN	NaN	NaN	NaN	NaN	NaN	NaN	NaN	NaN	NaN	NaN	NaN	NaN	NaN	NaN	NaN	NaN
SAM-H+LoRA [10]	95.55	94.77	93.04	93.13	90.94	73.29	70.56	73.73	59.15	80.71	81.09	69.99	78.62	93.99	87.74	88.04	82.27
U-SAM-H	NaN	NaN	NaN	NaN	NaN	NaN	NaN	NaN	NaN	NaN	NaN	NaN	NaN	NaN	NaN	NaN	NaN
U-SAM-H+LoRA	95.80	94.33	91.24	91.17	92.53	75.18	79.50	79.05	64.28	83.50	83.98	68.64	80.74	94.71	88.16	88.09	84.43

Supplementary Table 3

Comparisons of performance with variations of state-of-the-art medical segmentation methods on the WORD dataset. 'B' denotes that the model utilizes 'ViT-B', while 'H' indicates 'ViT-H'. 'LoRA' refers to the model implemented using Low-Rank Adaptation[16]. HFL:Head of Femur (L), HFR:Head of Femur(R). 'NaN' indicates that the value exceeds the evaluative capacity of our computational resources.

Category	Value	Count	Percentage (%)
T Stage	T1	1	5.0
	T2	5	25.0
	T3	11	55.0
	T4	3	15.0
N Stage	N0	10	50.0
	N1	6	30.0
	N2	4	20.0
Gender	Male	13	65.0
	Female	7	35.0

Supplementary Table 4

Detailed clinical information of the clinical test subset.

Method	Normal		Tumor		Mean	
	Dice (%)	IoU (%)	Dice (%)	IoU (%)	Dice (%)	IoU (%)
UCTransNet [22]	63.00	45.98	72.90	57.35	67.95	51.67
TransUnet-B [3]	60.74	43.62	70.65	54.62	65.70	49.12
SwinUnet-B [4]	63.32	46.32	72.63	57.02	67.97	51.67
TransUnet-L [3]	63.75	46.79	72.60	56.98	68.17	51.86
SwinUnet-L [4]	61.66	44.57	72.58	56.97	67.12	50.77
SAM-B [7]	60.95	43.83	71.00	55.04	65.98	49.44
SAM-B+LoRA [10]	57.57	40.42	70.70	54.68	64.14	47.55
U-SAM-B	65.72	48.94	72.84	57.28	69.28	53.11
U-SAM-B+LoRA	64.24	47.32	72.50	56.86	68.37	52.09
SAM-L [7]	61.68	44.59	72.46	56.81	67.07	50.70
SAM-L+LoRA [10]	59.46	42.31	70.73	54.71	65.09	48.51
U-SAM-L	64.58	47.69	73.55	58.17	69.06	52.93
U-SAM-L+LoRA	64.86	47.99	71.91	56.14	68.68	52.07
SAM-H [7]	NaN	NaN	NaN	NaN	NaN	NaN
SAM-H+LoRA [10]	60.05	42.91	70.72	54.70	65.38	48.80
U-SAM-H	NaN	NaN	NaN	NaN	NaN	NaN
U-SAM-H+LoRA	65.20	48.36	72.70	57.11	68.95	52.74

Supplementary Table 5
Comparisons of performance with variations of state-of-the-art medical segmentation methods on the CARE dataset. 'B' denotes that the model utilizes 'ViT-B', while 'H' indicates 'ViT-H'. 'LoRA' refers to the model implemented using Low-Rank Adaptation [16]. 'NaN' indicates that the value exceeds the evaluative capacity of our computational resources.

Area	Dice (%)	IoU (%)	NSD (%)
Normal	46.90	31.43	33.34
Tumor	58.87	43.59	19.98
Mean	52.88	37.51	26.66

Supplementary Table 6
3D U-Net [14] Segmentation Results. Segmentation performance of the 3D U-Net [14] model for normal and tumor areas, and overall mean.
