# Supplementary material for: Tuning vision foundation models for rectal cancer segmentation from CT scans
Source: Commun Med (Lond). 2025 Jul 1;5:256. doi: 10.1038/s43856-025-00953-0 (PMC12219254; doi:10.1038/s43856-025-00953-0)
Supplement: Supplementary file 2 — Description of Additional Supplementary Files [file 43856_2025_953_MOESM2_ESM.pdf]

Supplementary Data legends

Our Supplementary Data contains eight files. Based on "Supplementary Data 1.xlsx" to "Supplementary Data 7.xlsx", the numerical results in Table 3 and Figure 5 can be derived (Table 3 presents the comparisons of efficiency and accuracy between clinicians and our proposed U-SAM; Figure 5 shows the effect of skip-connections on U-SAM performance.).

Specifically, "Supplementary Data 1.xlsx" represents the accuracy of doctor group 1.
"Supplementary Data 2.xlsx" represents the accuracy of doctor group 2.
"Supplementary Data 3.xlsx" represents the accuracy of doctor group 3.
"Supplementary Data 4.xlsx" evaluates the efficiency of different categories of clinicians.
"Supplementary Data 5.xlsx" assesses the accuracy of the U-SAM model.
"Supplementary Data 6.xlsx" assesses the accuracy of the U-SAM/B model.
"Supplementary Data 7.xlsx" assesses the accuracy of the U-SAM/P model.
"Supplementary Data 8.xlsx" contains the raw data for Figure 5.
