# Supplementary material for: Tuning vision foundation models for rectal cancer segmentation from CT scans
Source: Commun Med (Lond). 2025 Jul 1;5:256. doi: 10.1038/s43856-025-00953-0 (PMC12219254; doi:10.1038/s43856-025-00953-0)
Supplement: Supplementary file 11 — Reporting Summary [file 43856_2025_953_MOESM11_ESM.pdf]

nature portfolio

Corresponding author(s): Shouhong Wan

Last updated by author(s): May 11, 2025

Reporting Summary

Nature Portfolio wishes to improve the reproducibility of the work that we publish. This form provides structure for consistency and transparency in reporting. For further information on Nature Portfolio policies, see our Editorial Policies and the Editorial Policy Checklist.

Statistics

For all statistical analyses, confirm that the following items are present in the figure legend, table legend, main text, or Methods section.

n/a	Confirmed	
☐	☒	The exact sample size (n) for each experimental group/condition, given as a discrete number and unit of measurement
☐	☒	A statement on whether measurements were taken from distinct samples or whether the same sample was measured repeatedly
☐	☒	The statistical test(s) used AND whether they are one- or two-sided<br>*Only common tests should be described solely by name; describe more complex techniques in the Methods section.*
☐	☒	A description of all covariates tested
☐	☒	A description of any assumptions or corrections, such as tests of normality and adjustment for multiple comparisons
☒	☐	A full description of the statistical parameters including central tendency (e.g. means) or other basic estimates (e.g. regression coefficient) AND variation (e.g. standard deviation) or associated estimates of uncertainty (e.g. confidence intervals)
☒	☐	For null hypothesis testing, the test statistic (e.g. F, t, r) with confidence intervals, effect sizes, degrees of freedom and P value noted<br>*Give P values as exact values whenever suitable.*
☒	☐	For Bayesian analysis, information on the choice of priors and Markov chain Monte Carlo settings
☐	☒	For hierarchical and complex designs, identification of the appropriate level for tests and full reporting of outcomes
☒	☐	Estimates of effect sizes (e.g. Cohen's d, Pearson's r), indicating how they were calculated

Our web collection on statistics for biologists contains articles on many of the points above.

Software and code

Policy information about availability of computer code

Data collection	The code is publicly available at: https://github.com/kanydao/U-SAM
Data analysis	We have provided detailed usage instructions for the code at: https://github.com/kanydao/U-SAM

For manuscripts utilizing custom algorithms or software that are central to the research but not yet described in published literature, software must be made available to editors and reviewers. We strongly encourage code deposition in a community repository (e.g. GitHub). See the Nature Portfolio guidelines for submitting code & software for further information.

Data

Policy information about availability of data

All manuscripts must include a data availability statement. This statement should provide the following information, where applicable:

- Accession codes, unique identifiers, or web links for publicly available datasets
- A description of any restrictions on data availability
- For clinical datasets or third party data, please ensure that the statement adheres to our policy

We have provided detailed instructions for obtaining and using the CARE and WORD data mentioned in our paper at: https://github.com/kanydao/U-SAM
Our proposed CARE dataset can be downloaded at: https://drive.google.com/file/d/1X_JTfD8Ch-IxmG5VHtKk_xGZT336Fl1Q/view

Human research participants

Policy information about studies involving human research participants and Sex and Gender in Research.

Reporting on sex and gender	In the CARE Dataset section of our paper, we provide a detailed description of the sex (biological attribute) characteristics within the dataset, including the number and proportion of patients by sex. All personal information has been removed during the data collection process to ensure privacy, and the dataset complies with relevant ethical guidelines, which are discussed in detail in the paper. In our experimental design, we did not make specific adjustments based on sex; therefore, the experimental results are applicable to the entire population of rectal cancer patients.
Population characteristics	In the CARE Dataset section of our paper, we provide a detailed description of the population characteristics. All samples included in the dataset are rectal cancer patients, and we have thoroughly discussed the corresponding inclusion criteria.
Recruitment	The participants in the study were from the First Affiliated Hospital of Anhui Medical University, Hefei, China. All personal information was removed during the data collection process to ensure privacy, and the dataset complies with relevant ethical guidelines. These ethical considerations and constraints are discussed in detail in the paper.
Ethics oversight	This study was approved by the Ethics Committee of the First Affiliated Hospital of Anhui Medical University (No. Quick-PJ 2023-13-34).

Note that full information on the approval of the study protocol must also be provided in the manuscript.

Field-specific reporting

Please select the one below that is the best fit for your research. If you are not sure, read the appropriate sections before making your selection.

☒ Life sciences ☐ Behavioural & social sciences ☐ Ecological, evolutionary & environmental sciences

For a reference copy of the document with all sections, see nature.com/documents/nr-reporting-summary-flat.pdf

Life sciences study design

All studies must disclose on these points even when the disclosure is negative.

Sample size	We summarized data from patients who underwent radical surgery for rectal cancer from January 2017 to June 2023. Samples were selected based on the inclusion criteria described in the paper, and we made extensive efforts to annotate the sample data. This process resulted in 398 annotated cases, with detailed descriptions available in the paper.
Data exclusions	During the construction of the rectal cancer dataset, we primarily focused on ensuring data quality. For example, we retained data with complete high-resolution CT scans of the rectum and dynamic enhanced CT scans of the pelvis while excluding cases without a clear pathological diagnosis of adenocarcinoma. Detailed inclusion and exclusion criteria for dataset construction can be found in the paper. In the evaluation and testing process of the dataset, no corresponding exclusions were made.
Replication	The experimental results are reproducible. All our collected datasets and the code used in the experiments have been made publicly available. The corresponding experimental results and the methods to obtain the pre-trained weights used can be found at: https://github.com/kanydao/U-SAM
Randomization	We conducted a random split of the CARE dataset into two subsets: 318 cases for training and 81 cases for testing.
Blinding	The investigators were blinded to group allocation during both data collection and analysis.

Reporting for specific materials, systems and methods

We require information from authors about some types of materials, experimental systems and methods used in many studies. Here, indicate whether each material, system or method listed is relevant to your study. If you are not sure if a list item applies to your research, read the appropriate section before selecting a response.

Materials & experimental systems

n/a	Involved in the study
☒	☐ Antibodies
☒	☐ Eukaryotic cell lines
☒	☐ Palaeontology and archaeology
☒	☐ Animals and other organisms
☒	☐ Clinical data
☒	☐ Dual use research of concern

Methods

n/a	Involved in the study
☒	☐ ChIP-seq
☒	☐ Flow cytometry
☒	☐ MRI-based neuroimaging
